# Carbon response of tundra ecosystems to advancing greenup and snowmelt in Alaska

**DOI:** 10.1038/s41467-021-26876-7

**Published:** 2021-11-25

**Authors:** JiHyun Kim, Yeonjoo Kim, Donatella Zona, Walter Oechel, Sang-Jong Park, Bang-Yong Lee, Yonghong Yi, Angela Erb, Crystal L. Schaaf

**Affiliations:** 1 Department of Civil and Environmental Engineering, Yonsei University, Seoul, Republic of Korea; 2 Department of Biology, San Diego State University, San Diego, CA USA; 3 Department of Animal and Plant Science, University of Sheffield, Sheffield, UK; 4 Department of Geography, University of Exeter, Exeter, UK; 5 Division of Atmospheric Sciences, KOPRI, Incheon, Republic of Korea; 6 Joint Institute for Regional Earth System Science and Engineering, University of California, Los Angeles, CA USA; 7 School for the Environment, University of Massachusetts Boston, Boston, MA USA

**Keywords:** Phenology, Ecological modelling

## Abstract

The ongoing disproportionate increases in temperature and precipitation over the Arctic region may greatly alter the latitudinal gradients in greenup and snowmelt timings as well as associated carbon dynamics of tundra ecosystems. Here we use remotely-sensed and ground-based datasets and model results embedding snowmelt timing in phenology at seven tundra flux tower sites in Alaska during 2001–2018, showing that the carbon response to early greenup or delayed snowmelt varies greatly depending upon local climatic limits. Increases in net ecosystem productivity (NEP) due to early greenup were amplified at the higher latitudes where temperature and water strongly colimit vegetation growth, while NEP decreases due to delayed snowmelt were alleviated by a relief of water stress. Given the high likelihood of more frequent delayed snowmelt at higher latitudes, this study highlights the importance of understanding the role of snowmelt timing in vegetation growth and terrestrial carbon cycles across warming Arctic ecosystems.

## Abstract (web summary)

The ongoing disproportionate increases in temperature and precipitation in the Alaska may alter the latitudinal gradients in greenup and snowmelt timings as well as carbon dynamics. With a broad range of datasets and model results, the authors show that the carbon response to early greenup or delayed snowmelt varies greatly depending upon local climatic limits.

## Introduction

The Arctic ecosystems are critical elements of the global carbon cycle and are greatly sensitive to ongoing warming (0.75 °C per decade during 1998–2012)^1^, which is more than twice faster than that for the rest of the globe. This warming has resulted in unprecedented changes across the region^2^, including spring greenup and snowmelt timings that are earlier by 4.3 and 5.5 days per decade, respectively^3,4^ (with large variation, up to 12–14 days per decade, depending on local environmental conditions and vegetation species). Most research has focused on analyzing the role of rising temperatures in earlier greenup timings; however, recent studies showed that persistent snow cover that lasts until temperatures are warm enough for greenup (hereafter refer as “delayed snowmelt”) results in delaying greenup, which exerts as strong a control as warming temperatures do, but in the opposite direction^5,6^. Unlike greenup, which is primarily driven by spring temperature, snowmelt timing is determined by two covarying factors: temperature and precipitation^7^. Notably, the amount and frequency of winter precipitation are projected to increase by as much as 25% in the Arctic^8,9^, where the snowfall fraction of precipitation may change nonlinearly with respect to local warming^10^. Hence, across the region, changes in snowmelt timing may become more variable over time than advances in greenup with rising temperatures. Therefore, understanding how the greenup and snowmelt timings are changing relative to one another and its implication for vegetation growth and the carbon cycle of tundra ecosystems across the Arctic region is critical.

**Fig. 1:**
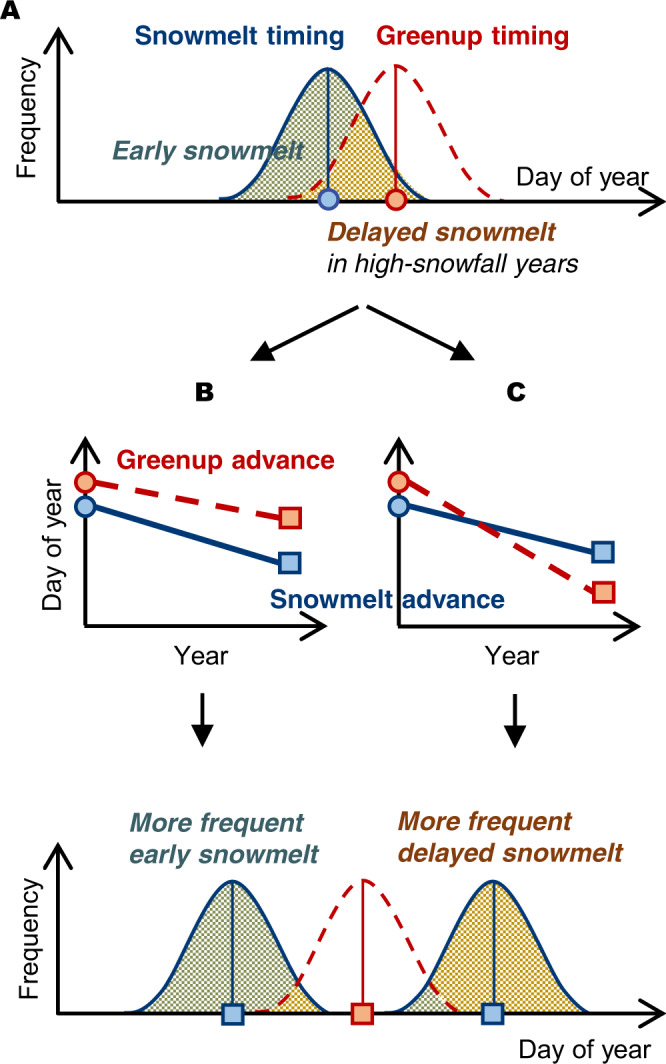
Schematic illustration of long-term trends of greenup and snowmelt timings and the resultant effects. **A** In a present-day ecosystem, snow cover usually melts (blue circle) before greenup occurs (red circle) with interannual variations (blue and red distributions for snowmelt and greenup timings, respectively), and snowmelt is occasionally delayed when there is high snowfall (chance of early snowmelt and delayed snowmelt events in green and brown areas, respectively). The relative advance of the two timings (snowmelt in the blue solid line and greenup in the red dashed line) decide whether there will be **B** more frequent early snowmelt events or **C** more frequent delayed snowmelt events (i.e., suspending greenup from the meteorologically possible timing).

Depending on how snowmelt and greenup timings change respectively, vegetation growth and ecosystem dynamics will be affected by more frequent early or delayed snowmelt timing. For example, those ecosystems that occasionally experience delayed snowmelt in high-snowfall years (Fig. 1A) may be increasingly affected by early snowmelt if snowmelt timing advances faster than greenup (Fig. 1B). By contrast, the direct impact of delayed snowmelt on vegetation growth (i.e., greenup delay) and, in turn, on the carbon cycle would be locally escalated in some ecosystems where greenup is expected to advance more rapidly than snowmelt (Fig. 1C). If the effect of early or delayed snowmelt timing is not separated from that of warming-driven early greenup, the temperature sensitivity of vegetation to ongoing Arctic warming may be underestimated, which may lead to a misrepresentation of ecosystem responses to projected climate change. For example, the higher carbon uptake with earlier snowmelt timing^11^ may not be directly attributed to earlier snowmelt but could be a result of a warm spring.

While the effect of snowmelt timing on the hydrological regime has been studied extensively using both field data and model simulations due to the importance of snowmelt runoff in water resource management^12,13^, investigations of the effect of changing snowmelt timing on carbon dynamics mostly relied on data analysis, with little consensus on the direction and magnitude of the implications. Several studies showed that carbon uptake was greatly enhanced by earlier snowmelt, which often accompanied high spring temperatures^11,14–16^. In contrast, others presented weak correlations between earlier snowmelt and carbon uptake^17–19^ or even negative ones (e.g., frost-drought damage^20^). Meanwhile, some studies suggested that vegetation greenness in water-limited ecosystems increased with higher snow accumulation (which often relates to delayed snowmelt)^21–24^, whereas others showed decreases in reproductive success due to the shortened growing season (GS) caused by delayed snowmelt^25^. However, such estimates based on data analysis inevitably include the coupled effects of the covarying factors (i.e., greenup and snowmelt timings varying along with meteorological changes). One study applied a process-based model and showed that local hydrological conditions affected the response of Arctic ecosystems (e.g., net carbon exchange, snowpack and permafrost depth at mixed tundra and fen sites) to meteorological variation^26^. However, the net effect of snowmelt timing changes was not investigated in the study, as snow melted earlier in warm years and later in cool years during the 4-year study period. Therefore, extensive analysis using both data and process-based models is still required to obtain a comprehensive understanding of carbon response to snowmelt timing changes under various hydrometeorological conditions, which is essential for more accurate predictions of Arctic ecosystems under changing climates.

Here, we investigated the controls of early greenup and delayed snowmelt on vegetation growth and the carbon cycle of Arctic tundra, using both a process-based model (Ecosystem Demography model version 2, ED2^27,28^) and observational datasets (flux tower measurements and remote sensing products) at seven flux tower sites in Alaska, USA, during 2001–2018 (Fig. S1A and Table S1). As temperature and precipitation vary differentially with respect to latitude, the types and degrees of climatic limits on ecosystem processes are expected to vary across the study sites^29^. We hypothesize that the impact of early greenup and delayed snowmelt would differ greatly according to local climatic limits (i.e., strong/moderate temperature and/or water) and also varies widely throughout the GS. First, we estimated the long-term trends of greenup and snowmelt timings at each study site using Moderate Resolution Imaging Spectroradiometer (MODIS) phenology and snow cover data. A case study was conducted at three sites (US-Atq, US-EML, and US-BZF), where flux data are available for longer than 5 years, to evaluate the changes in temporal patterns and amount in the flux due to delayed snowmelt. Based on the ED2 results implemented with the newly developed phenology module at the seven study sites, we investigated the net and lagged effects of changes in snowmelt timing (early and delayed, respectively) on vegetation growth and carbon uptake. Finally, we further analyzed the net effects of early greenup and delayed snowmelt throughout the GS for tundra ecosystems under different climatic limits.

## Results and discussion

### Climatic gradient and greenup and snowmelt timing trends

**Fig. 2:**
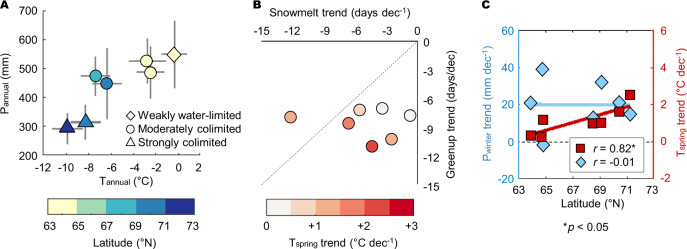
Latitudinal gradients in climatic conditions and greenup and snowmelt timings. **A** Annual mean temperature (T_annual_) and annual total precipitation (P_annual_) at each site (2001–2018 means in symbols and one std. dev. in lines) with latitude (in color) and climatic limits (see Methods—Study sites). **B** Long-term trends of snowmelt and greenup timings at each site (in circles, Fig. S3) and the spring mean temperature trend (T_spring_, Apr.–Jun., in color). **C** Correlations of long-term trends of T_spring_ and winter precipitation (P_winter,_ Jan.–May) with latitude.

We analyzed climatic variables (i.e., temperature, precipitation, and radiation) across the seven study sites (see Methods—Study sites) and found that the climatic conditions primarily varied with latitude. Annual temperature and precipitation were lower at higher latitudes than at lower latitudes (*p* < 0.05, Fig. 2A and S1B), while no such latitudinal gradient was identified for annual radiation (*p* = 0.75, not shown). These climatic gradients thereby resulted in different types and degrees of climatic limits with latitude (Fig. S2 and Table S2, see Methods—Study sites). Vegetation growing days at the two northernmost sites (US-Beo and US-Atq) were strongly limited by both temperature and water, with a 43–51% reduction due to temperature and 38–43% due to water. Moreover, the net ecosystem exchange (NEE) was significantly responsive to the temperature and vapor pressure deficit (VPD), with mostly *P* < 0.001 (hereafter referred to as “strongly colimited sites”). Meanwhile, vegetation growing days at lower latitudes (US-Hva, Us-Ivo, KOPRI, and US-EML) were moderately colimited by temperature and water by 19–27% and 17–30%, respectively. Therefore, variations in the carbon flux at these sites were significantly driven by the temperature and VPD, by either *P* < 0.01 or *P* < 0.05 (hereafter referred to as “moderately colimited sites”). The US-BZF site, one of the southernmost sites, was hardly limited by temperature (8%) and weakly limited by water (19%), and its carbon flux variation was more accounted for by VPD (*P* < 0.001) than by temperature (*P* < 0.05) (hereafter referred to as “weakly water-limited site”). The radiation limit on vegetation growing days was weaker than the limits imposed by temperature and water, which was estimated as 14–16% over the study sites during the study period, and its control on NEE was mostly significant at the northernmost sites (*P* < 0.001). In addition, we found that the coupled effects of the meteorological variables on the carbon flux were also significant.

We analyzed MODIS snow cover and phenology data (see Methods—MODIS) and found strong latitudinal gradients in snowmelt and greenup timings across the study sites (*p* < 0.05, Fig. S3B)^30,31^. In general, both snowmelt and greenup timings occurred earlier at the lower latitudes, where the timings were 111–161 and 125–166 day of year (DOY), respectively, during 2001–2018 (std. dev. of 6 days at each site on average). Such latitudinal gradients were also found in their drivers, i.e., the spring mean temperature (T_spring_, Apr.–Jun., *p* < 0.01, Fig. S1B) and winter precipitation (P_winter_, Jan.–May, *p* < 0.10). Meanwhile, we found no elevational effect on snowmelt and greenup timings or on climatic conditions across the study sites (*r* = −0.25 and −0.50, respectively, with *p* > 0.05). At the seven study sites over Alaska, both snowmelt and greenup timings advanced during 2001–2018 (Fig. 2B and S3). The mean rates of advance were 5.0 and 8.4 days per decade for snowmelt and greenup, respectively (std. dev. of 3.7 and 1.5, respectively). The long-term trends of greenup timing were significant at one site (*p* < 0.05), whereas the trends of snowmelt timing were not significant at all sites. The rates of advance of greenup timing were higher than those of snowmelt timing for most of the study sites, which indicates that there will be a greater likelihood of greenup occurring close to snowmelt timings over the coming years, resulting in frequent delayed snowmelt events (as in Fig. 1C). We also found that the advance trends of greenup timing could be greatly accounted for by the increasing trends in T_spring_ across the study sites (*r* = −0.75, *p* < 0.05, Fig. S1C), but the advance trends of snowmelt timing were not (*r* = −0.33, *p* > 0.05; note that a negative value indicates an earlier timing). Instead, the advance trends in snowmelt timing were explained by the decreasing trends in P_winter_ during the study period (*r* = 0.91, *p* < 0.01, Fig. S1C). There was also a significant correlation between the increasing trends in T_spring_ and latitude, i.e., faster increases at higher latitudes (*r* = 0.82, *p* < 0.05, Fig. 2C); however, no such correlation was found for the P_winter_ trends with latitude across the study sites. As a result of such disproportionate changes in T_spring_ and P_winter_ with latitude, interference of greenup onset by snow cover may become more evident at higher latitudes (i.e., more frequent delayed snowmelt), whereas delays in snowmelt events are likely to occur only with extreme snowfall at lower latitudes. Meanwhile, there was no significant long-term trend in snowpack or dormancy timing at these study sites (*p* > 0.05, not shown).

It should be noted that the long-term trends in greenup and snowmelt timings based on the MODIS data may include uncertainties associated with remote sensing data, such as methodological differences for phenological transition detection^32,33^, chosen remote sensing indices^30^, a weak signal-to-noise ratio for remote sensing at high latitudes (e.g., low sun angle, low amplitude of vegetation index^34^, and persistent overcast^35^), and a spatial discrepancy between the area of interest and the spatial resolution of the satellite instruments. Qualitative assessment of transition timings based on remote sensing data at high latitudes has long been a challenging task due to the rare availability of in situ data and the heterogeneous landscapes in the Arctic and boreal regions at the remote sensing data scale^36^. Therefore, prior to estimating long-term trends using MODIS data, we assessed the spatial representativeness of the landscape of PhenoCam sites^37^, National Climatic Data Center (NCDC) stations (Table S3), and study sites at the MODIS spatial scales (1 × 1 and 3 × 3 pixel windows, respectively, see Supplementary Note) and showed that the MODIS greenup and snowmelt timings agreed well with ground data at a spatially representative scale (i.e., a 1 × 1 pixel window, see Supplementary Note). Our results highlight that it is critical to consider landscape heterogeneity when using remote sensing data for site-level analysis at high latitudes^36^, which should be followed with an increasing amount of long-term ground data (e.g., PhenoCam^37^).

### Case study: flux data analysis

**Fig. 3:**
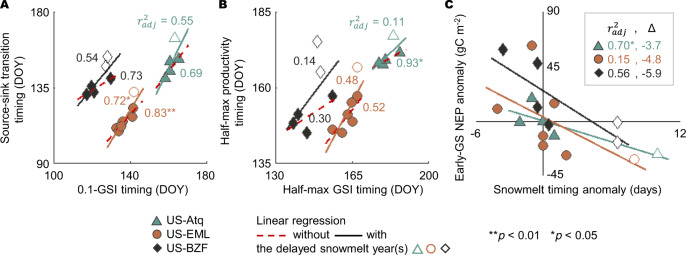
Changes in flux timing and magnitude due to delayed snowmelt. At three sites (US-Atq, US-EML, and US-BZF), **A**, **B** correlations between flux timings (source-sink transition timing and half-max productivity timing) and meteorological timings (0.1-GSI timing and half-max GSI timing) with and without the delayed snowmelt year(s) (empty markers), and **C** decreases in the early growing season net ecosystem productivity (early-GS NEP) by snowmelt timing delay (Δ).

At three sites (US-Atq, US-EML, and US-BZF) where flux data were measured for more than 5 years, we calculated two meteorological timings (0.1-GSI timing and half-max GSI timing) and two flux-threshold timings (source-sink transition timing and half-max productivity timing) (see Methods—Case study). By comparing these flux timings against meteorological timings, we found that delayed snowmelt greatly affected the local carbon cycle by suspending the onset of vegetation carbon uptake and also decreasing the amount of carbon sequestered during the early GS (Fig. 3). The lower agreement between the flux-threshold timings and the meteorological timings with the year(s) when the snowmelt was delayed (Fig. 3A, B) indicates that dominant meteorological control on vegetation carbon uptake was disrupted by delayed snowmelt. We also estimated that delaying snowmelt timing by ten days would reduce the net ecosystem productivity (NEP) by 36.7–59.0 g C m^−2^ during the early GS across the three sites (Fig. 3C). However, it is important to note that this data-based analysis is inevitably restricted as (1) the number of data points was limited (i.e., *N* = 5–7 in this study, with one or two delayed snow event(s)) and (2) the interannual meteorological variation resulting in changes in flux cannot be eliminated, rendering it infeasible to investigate the lagged effects of early or delayed snowmelt over the following GS. These restrictions call for the implementation of a process-based model, ED2 in this study, to further analyze how changes in greenup and snowmelt timings affect the regional carbon cycle of the Arctic region under various climatic limits.

### Effects of early and delayed snowmelt

We further explored the net and lagged effects of early and delayed snowmelt on vegetation growth and the carbon cycle using a process-based ecosystem model, ED2, during 2001–2018. A newly developed phenological index, the snowmelt-growing season index (SGSI, see Methods—SGSI), was incorporated into ED2, followed by calibration and validation (see Methods—ED2). The SGSI acts as a threshold that postpones vegetation greenup onset if the snow has not melted, even when the meteorological conditions for greenup were satisfied. We applied a least-squares linear regression between the snowmelt timing variation days and the deviation in each process (see Methods—Correlation analysis).

**Fig. 4:**
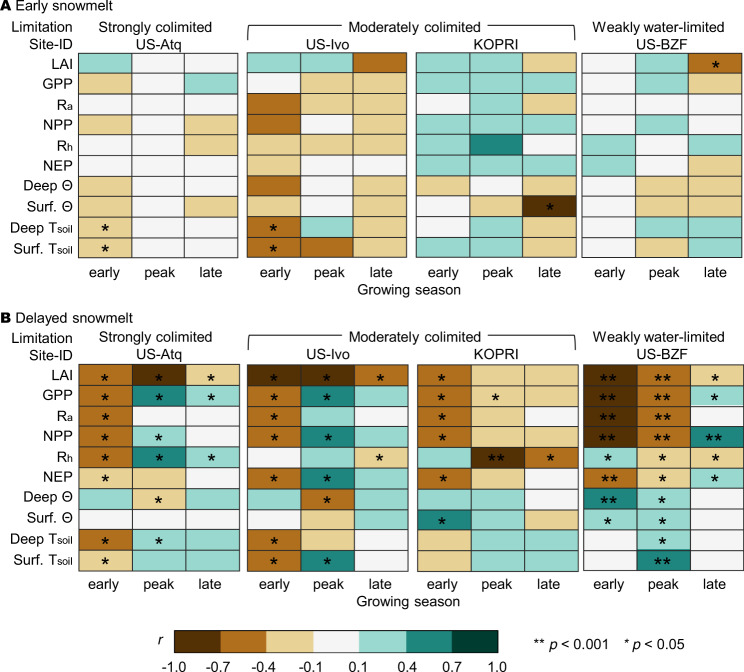
Seasonal effects of early or delayed snowmelt timing on ecosystem processes. A least-squares linear regression was applied for the years when meteorological conditions were similar (see Methods—Correlation analysis). **A** Pearson correlation (*r*) between early snowmelt days (deviation in snowmelt timing each year from the mean snowmelt timing) and the changes in each ecosystem process (leaf area index, LAI; gross primary productivity, GPP; autotrophic respiration, R_a_; net primary productivity, NPP; heterotrophic respiration, R_h_; net ecosystem productivity, NEP; deep and surface soil moisture, Θ; deep and surface soil temperature, T_soil_) from the ED2 simulation for each year (i.e., the difference between the seasonal mean and the mean value over the years), and **B** Pearson correlation (*r*) between the delayed snowmelt days and the difference between the ED2 modeled results with GSI and SGSI. Other sites are presented in Fig. S4.

Our results showed that the increase in snow-free days by early snowmelt had little effect on vegetation growth and carbon fluxes (Fig. 4A and Fig. S4A) when meteorological conditions were similar, leading to little change in greenup timing. The leaf area index (LAI) increased with early snowmelt at most of the study sites during the early GS, but not significantly (*p* > 0.05). Decreases in the LAI were found during the late GS, with a significant decrease at the weakly water-limited site. However, decreases in carbon uptake (i.e., gross primary productivity, GPP) and plant respiration (i.e., autotrophic respiration, R_a_) were not significant, even with the significant reduction in the LAI. Soil moisture decreased at most sites as a result of the reduced winter precipitation, which resulted in early snowmelt. Notably, the soil temperature during the early GS was significantly decreased by early snowmelt at the higher latitude sites (*p* < 0.05 at the two strongly colimited sites and two moderately colimited sites), as the soil was exposed to cold air, as shown in snow removal experiments^20^. At the sites where the soil temperature significantly decreased with early snowmelt, heterotrophic respiration (R_h_) was reduced as well (yet *p* > 0.05 at most sites) due to soil temperature and moisture limiting microbial activity^18^. On the other hand, soil temperatures at the lower latitude sites increased nonsignificantly with early snowmelt (*p* > 0.05). Our results showed that when constraining meteorological conditions and greenup timing, early snowmelt itself had little effect on early GS vegetation productivity^18^, but its effect lasted until the late GS depending on local hydrometeorological conditions (i.e., water and temperature limitations)^38^.

We found that delayed snowmelt affected both aboveground and belowground processes to various extents (Fig. 4B and Fig. S4B). First, as the leaf onset was physically impeded by delayed snowmelt, the LAI was reduced during the early GS regardless of the site climatic limits (*p* < 0.05 at all study sites), which lasted even until the late GS at most sites. Consequently, the GPP and R_a_ decreased significantly at all sites during the early GS. During the peak and late GS, however, the GPP and R_a_ increased even with an LAI reduction at two sites (one strongly colimited site and one moderately colimited site at a high latitude). Such decoupling indicates strong meteorological controls on vegetation physiological activities (i.e., carbon uptake and respiration) at higher latitudes, which is even greater than the controls by leaf areas (i.e., a physical measure of the photosynthetic active area). The increases in soil moisture with delayed snowmelt were most evident at the weakly water-limited site (US-BZF, *p* < 0.05 during the early and peak GS), reflecting extra water availability in soils where vegetation carbon uptake was not constrained by a lack of water. Meanwhile, soil temperatures decreased in response to delayed snowmelt during the early GS at all sites, even significantly at four higher latitude sites. This indicates that the not-yet-melted snow cover insulated the soil from the air, which was warm enough for vegetation greenup. The increases in soil temperature during the following peak GS were probably attributable to the increased incoming radiation input resulting from the decreased leaf area. We found that changes in R_h_ followed the soil temperature changes at the higher latitude sites; however, at the lower latitude sites (at KOPRI and US-BZF, where water is more limiting than temperature), the R_h_ response to the soil temperature change diverged (i.e., a significant decrease when soil moisture increased with delayed snowmelt during the peak GS). This result of a high moisture-limiting R_h_ aligns with the previous findings in that the response of R_h_ to soil temperature greatly depends on site conditions; R_h_ is sensitive to soil temperature when the site is strongly limited by temperature, whereas soil moisture becomes more critical when the site is less or not constrained by temperature^39^. As a result of all these carbon uptake and respiration dynamics, the negative effect of delayed snowmelt (i.e., decreases in the NEP during the early GS) was alleviated toward the peak or late GS as water stress was relieved by increased water availability.

### Comparison of the net effects of early greenup and delayed snowmelt

**Fig. 5:**
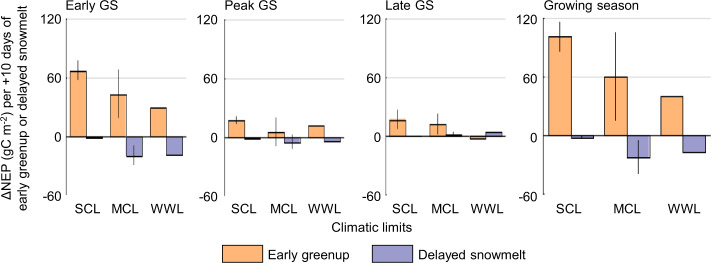
Net ecosystem productivity (NEP) changes due to early greenup and delayed snowmelt under each climatic limit. The NEP change (ΔNEP) per 10 days of early greenup or delayed snowmelt with each climatic limit (strongly temperature and water-colimited, SCL; moderately colimited, MCL; weakly water-limited, WWL). The mean value of the sites with the same limit is represented as a bar, with one standard deviation indicated by the vertical line (there is only one weakly water-limited site, therefore no standard deviation line).

The net and lagged effects of early greenup were estimated from a linear regression between greenup timing deviation and the seasonal deviation of NEP at each site and compared with the effects of delayed snowmelt, based on the ED2 results during 2001–2018. Our analysis showed that the net effects of the two controls, warming-driven early greenup and delayed snowmelt, were greatly different depending on site climatic limits as well as the season (Fig. 5).

During the early GS, the NEP increase due to early greenup was distinct at the strongly colimited sites (67.2 g C m^−2^ per 10 days of early greenup on average) compared to the moderately colimited and weakly water-limited sites (42.7 and 29.7 g C m^−2^, respectively). This indicates that the carbon uptake of temperature-sensitive vegetation at the higher latitude sites was amplified due to the longer greenup period with warmer temperatures. However, at the lower latitude site (i.e., the weakly water-limited site), the control of early greenup was approximately similar to that of delayed snowmelt during the early GS (18.5 g C m^−2^ per 10 days of delayed snowmelt). Furthermore, the NEP decreases due to delayed snowmelt at the strongly colimited sites were ~20% of those at the moderately colimited and weakly water-limited sites. This implies that the water stress at the strongly colimited sites was, to some extent, alleviated by delayed snowmelt (i.e., increased water input), resulting in smaller NEP decreases. However, we did not find any significant increases in peak GS vegetation growth attributable to water stress relief, as shown in previous studies^24,40^. The ED2-based NEP decrease during the early GS due to delayed snowmelt at the case study sites (4.4, 18.5, and 23.2 g C m^−2^ per 10 days at the US-Atq, US-BZF, and US-EML) was ~12–48% of the estimate from the case study using flux tower data (36.7, 59.0, and 48.0 g C m^−2^ per 10 days, Fig. 3C). The difference between the two analyses may be attributed to the experimental setup; the case study tends to highlight the extreme event(s), but the model-based analysis included various levels of delayed snowmelt events. The estimates from the case study may also include flux responses to interannual meteorological variations. The climatic limits on the NEP response to the two controls, early greenup and delayed snowmelt, became more apparent at the GS scale. The NEP increases with warming-driven early greenup were approximately two times greater at the strongly colimited sites (101.1 g C m^−2^ per 10 days of early greenup on average) than at the other sites, whereas the NEP decrease with delayed snowmelt at the strongly colimited sites was ~10% of that at the weakly water-limited and moderately colimited sites (2.1 g C m^−2^ per 10 days of delayed snowmelt).

### Concluding remarks

The Arctic ecosystems are experiencing unprecedented climate changes; therefore, understanding the net effect of individual climatic controls is essential for a better assessment of ecosystem sensitivity to changing climates. Our analysis of meteorological and remote sensing data at seven tundra study sites in Alaska reveals strong latitudinal gradients in climatic conditions, greenup and snowmelt timings, and their changes. These results suggest that delayed snowmelt timing may become more frequent at higher latitudes while occasional delays in snowmelt timing will continue at lower latitudes with extreme winter precipitation events. The model simulation analysis shows that the control of delayed snowmelt on carbon dynamics is as strong as warming-driven early greenup during the early GS and also has lasting effects throughout the GS. Our results also indicate that the response of the Arctic tundra to early greenup or delayed snowmelt depends greatly on local climatic limits. Amplification of increases in carbon uptake due to warming-driven early greenup was evident at the strongly colimited sites, where water stress relief with delayed snowmelt was also apparent. The results presented in this study imply that the level of carbon uptake in high latitude regions will be greatly overestimated if only early greenup with warming temperatures is considered without properly accounting for the implications of delayed snowmelt on vegetation growth and ecosystem dynamics. At lower latitudes, the control of delayed snowmelt should also be accounted for to avoid underestimating the effect of warming on NEP changes. This study highlights the importance of snowmelt timing for the phenology of the tundra ecosystem, as well as its impact on vegetation growth and carbon dynamics under the climatic limits for better understanding of carbon exchange between the biosphere and atmosphere in the Arctic region under ongoing climate change.

## Methods

### Study sites: site description and climatic limit

In this study, we focused on seven flux tower sites located in Alaska, United States, including six AmeriFlux sites and one site of the Korea Polar Research Institute (KOPRI) (Fig. S1A, Table S1). Over the seven study sites, the annual mean temperature was between −10.09 and −0.55 °C and the annual total precipitation ranged from 287 to 540 during 2001–2018 based on the North American Regional Reanalysis (NARR^41^, 0.3-degree resolution every 3 h). The annual mean temperature increased from 2001 to 2018 at all sites, with rates between 0.5 and 2.2 °C per decade (*p* < 0.05 at five sites), whereas there were no such significant trends in the annual total precipitation (*p* > 0.05 at all sites). Our study sites were mostly dominated by wet sedges, grasses, moss, lichens, and dwarf shrubs. For example, the dominant plants at the US-Atq site (at a higher latitude) are herbaceous sedges (*Carex aquatilis, Eriophorum russeolum*, and *Eriophorum angustifolium*) and shrubs (*Salix rotundifolia*), with abundant mosses (*Calliergon richardsonii* and *Cinclidium subrotundum*) and lichens (*Peltigera* sp.)^11^. At the KOPRI site (at a lower latitude), mosses (*Sphagnum magellanicum, Sphagnum angustifolium*, and *Sphagnum fuscum*), lichens (*Cladonia mitis, Cladonia crispata*, and *Cladonia stellaris*), and tundra tussock cottongrass (*Eriophorum vaginatum*) are abundant^39^. The active layer thickness is between 0.33 and 1.0 m, according to field data and radar-based estimates.

Climatic limits imposed by temperature, water, and radiation were quantified following Nemani et al.^42^ at each site during the GS between 2001 and 2018 (Fig. S2) using the NARR data. In this study, we defined the GS as from May to Oct., early GS as between May and Jun., peak GS as between Jul. and Aug., and late GS as between Sep. and Oct. For a temperature limit scalar, the monthly mean temperature from −5  to 5 °C was linearly scaled between 100% (i.e., no growth) and 0% (i.e., no reduction in growing days). The monthly ratio of precipitation to potential evapotranspiration (PET by the Priestley-Taylor method^43^), ranging between 0 and 0.75, was linearly scaled from 100 to 0% as a water limit scalar. A radiation limit scalar was estimated as a 0.5% reduction in growing days for every 1% increase in monthly cloudiness above the 10% threshold (monthly cloudiness (*n*) was estimated^44^ as $R={R}_{0}(1-0.75{n}^{3.4})$, where *R* and *R*_0_ are the monthly mean incoming radiation and clear-sky radiation^45^, respectively).

The carbon flux response to climatic variations at each site was further analyzed using a forward stepwise multiple regression analysis^11^ between the NEE and meteorological variables (temperature, PAR, and VPD) using tower data during the GS. Interaction terms among the variables are also included to consider the convolved effects of the variables (Eq. (1)).

$${Y}_{{{NEE}}}= {\beta }_{0}+{\beta }_{1}{X}_{{{T}}}+{\beta }_{2}\,{X}_{{{VPD}}}+{\beta }_{3}{X}_{{{PAR}}}+{\beta }_{4}{X}_{{{T}}}* {X}_{{{VPD}}}+\ldots$$

1 $$\qquad\;\;\; {\beta }_{5}{X}_{{{T}}}* {X}_{{{PAR}}}+{\beta }_{6}{X}_{{{VPD}}}* {X}_{{{PAR}}}+{\beta }_{7}{X}_{{{T}}}* {X}_{{{VPD}}}* {X}_{{{PAR}}}$$

where *Y*_NEE_ is the daily average NEE (µmol m^−2^ s^−1^), and *X*_T_, *X*_VPD_, and *X*_PAR_ are daily average air temperature (°C), VPD (ha), and PAR (µmol Photon m^−2^ s^−1^), respectively. Regression coefficients (${\beta }_{0},\ldots ,{\beta }_{7}$), standard errors, significance (*P*-value), and *R*^2^ value of the final regression model are summarized in Table S2.

### MODIS: long-term trends of snowmelt and greenup timings

We collected the gridded MODIS snow cover (MOD10A1.V006^46^ at a 500-m resolution every day) and phenology (MCD12Q2.V006^34^ at a 500-m resolution yearly) from the NASA Earthdata (https://earthdata.nasa.gov/). We estimated the snowmelt and snowpack timings at each site as the date when a logistic fit to the MODIS snow cover (quality flags of good and best) passed 0.1 each year. We rejected those snowmelt timings when the gaps in the daily MODIS snow cover were longer than 2 weeks around the time of the snowmelt event. The greenup and dormancy timings with a quality flag of best were taken from the MODIS phenology. Based on the spatial representativeness assessment (see Supplementary Note), we decided to use the snowmelt timing and greenup timing within a 1 × 1 pixel window.

The significance of the long-term trends in greenup and snowmelt timings at each site was determined by Spearman’s rho and Mann-Kendall tests (Fig. S3). We further estimated the 95% confidence intervals of the trends from 3000 timing sets generated by bootstrap resampling from a normal distribution^47^ (mean equal to each greenup or snowmelt timing with three standard deviation set to 10 or 6.6 days, respectively, i.e., the root-mean-squared values between the ground data-based estimates and MODIS values in a 1 × 1 pixel window, Figs. S9 and S10).

### SGSI: snowmelt-growing season index

Growing season index (GSI)^48^ is one of the novel phenology models^49^ and has been widely applied for the phenological representations of various ecosystems^50,51^. GSI is a product of three indices of climatic variables (Eq. (2), Fig. S5): daylength, VPD, and growing-degree-days (GDD)^52^. As a phenological measure for a given meteorological condition, we calculated the daily GSI for spring (from Jan. 1 to Jul. 31) and fall (from Aug. 1 to Dec. 31), respectively. For the spring-GSI, GDD is the degree sum when the daily mean temperature rises above −5 °C after Jan. 1. For the fall-GSI, GDD is the degree sum when the daily mean temperature falls below 20 °C after Aug. 1. We then revised the GSI by multiplying it by a snowmelt index (*iS*) and referred to this as the snowmelt-GSI (SGSI, Eq. (3), Fig. S5). This guarantees that vegetation greenup does not start unless snow is melted, even if the meteorological conditions are sufficient to trigger leaf-out. The *iS* was estimated to be 0 when the snow cover fraction (*snowfac* variable^53^ in ED2) was above 0.1 and 1 otherwise.

2 $${{{GSI}}}={{iX}}_{1} \times {{iX}}_{2} \times {{iX}}_{3}$$

3 $${{{SGSI}}}={{{GSI}}} \times {iS}$$

where *iX* (*X*_1_, *X*_2_, and *X*_3_ represent daylength, VPD, and GDD, respectively) is 0 (*X* ≤ *X*_min_), 1 (*X* ≥ *X*_max_), and (*X* *−* *X*_min_)/(*X*_max_ − *X*_min_) otherwise. *X*_max_ and *X*_min_ are the maximum and minimum thresholds of each index, respectively. For the spring-GSI, *X*_min_ was calculated as the minimum value among the values on the greenup day (from MCD12Q2.V006) for the study period of 2001–2018 at each study site, and *X*_max_ was calculated as the minimum value among the values on the maturity day. For the fall-GSI, similarly, *X*_min_ was the minimum value for the dormancy timings, and *X*_max_ was the minimum value for the senescence timings. We incorporated GSI (or SGSI) into ED2 by multiplying it to the optimal value of leaf biomass on the day, where it operates as an upper limit of the leaf biomass.

In this study, it was assumed that phenological stages are driven by meteorological conditions, not by other factors (e.g., no assumption of fixed phenological periods^6,54,55^). The development of a robust phenological model for the tundra ecosystem would be enabled by an increasing amount of ground-based phenology data (e.g., PhenoCam data^37^).

### Case study: flux data analysis

There were three sites where flux data is available for >5 years in Alaska; US-Atq site (flux data during 2004–2008 with delayed snowmelt in 2005), US-EML site (flux data during 2009–2017 with delayed snowmelt in 2017), and US-BZF (flux data during 2012–2018 with delayed snowmelt in 2017 and 2018). We first calculated two timings that are related to the meteorological conditions (0.1-GSI timing and half-max GSI timing^51^, Fig. S6) using the NARR data. The 0.1-GSI timing and half-max GSI timing were calculated on the day when the GSI passed 0.1 and the half-max value (i.e., 0.5), respectively, each year. To calculate two timings regarding the flux seasonal profile^51^ (source-sink transition timing and half-max productivity timing, Fig. S6), we used 30-min gap-filled FLUXNET2015^56^ data (NEE and GPP; quality flags of measured or good) at the US-Atq site to calculate daily NEP (i.e., negative NEE) and daily GPP. At the US-EML and the US-BZF sites, we applied an open-source code called ONEFlux (Open Network-Enabled Flux processing pipeline for eddy-covariance data)^57^ using the ERA5 data (European Centre for Medium-Range Weather Forecast Reanalysis v5^58^) which was downscaled with a quantile mapping method^59^. Using the gap-filled 30-min NEP and GPP data from the ONEFlux, we calculated the corresponding daily values and fitted a smoothing spline to the daily NEP and the daily GPP each year. The source-sink transition timing was defined as the day when the smoothing spline of the daily NEP passed zero in each year. The half-max productivity timing was set to the day when the smoothing spline of the daily GPP passed the half-max value in that year^51^.

Further, we investigated whether the delayed snowmelt altered the relationships between meteorological conditions and the flux-threshold timings at each site based on (1) the correlation between the 0.1-GSI timing and the source-sink transition timing and (2) the correlation between the half-max GSI timing and the half-max productivity timing.

### ED2: model implementation

We used NARR data^41^ (0.3-degree resolution every 3 h; temperature, precipitation rate, pressure, v- and u-wind speed, downward longwave and shortwave radiation flux, and relative and specific humidity) for single-point ED2 implementation at each study site from 2001 to 2018. Vegetation structure (LAI, leaf and structural biomass, diameter at breast height, and population density) was initialized for each site by using the maximum annual LAI of cold-adapted shrubs and Arctic C3 grass from the Ent Global Vegetation Structure Dataset v1.0b (Ent-GVSD v1.0b) with the allometric equations in ED2. Ent-GVSD v1.0b provides plant functional types (from the MODIS land cover, MCD12C1.V005^60^) and maximum annual LAI values (from the MODIS LAI, MOD15A2.V004^61,62^) in subgrid cover fractions. We did not use canopy heights from Ent-GVSD v1.0b because of the absence of trees at the study sites. Soil texture (the ratio of sand:silt:clay) was set following the Harmonized World Soil Database v1.1^63^ of the Food and Agriculture Organization of the United Nations (UN FAO). Soil carbon was initialized using the UN FAO Global Soil Organic Carbon Map^64^, and soil nitrogen was estimated using the soil C/N ratio of moist tundra (mean: 18.4)^65^.

The prior distribution of each key variable was based on previous studies (Table S4), and 10,000 parameter sets were randomly generated from the prior distributions (the so-called Monte Carlo method). The best parameter set was selected based on statistical measures (*r*^2^ and root-mean-squared error) when compared to the data at the US-Atq site, i.e., NEP flux data for 2004–2006 and MODIS LAI data for 2003–2010 (MCD15A3H.V006^66^ at a 500-m resolution every 4 days) (Table S5). We then validated the performance of ED2 with this best parameter set by focusing on key ecosystem processes, such as NEP, ecosystem respiration, soil temperature, snowmelt timing, greenup timing, and the LAI at all sites (Table S5). The ED2 LAI was overestimated by 0.15–0.16 compared to the field-measured LAI values (Jul.–Aug. in 2006 at Barrow^67^ and Jun.–Aug. in 1996 at Toolik^68^).

It is worth noting that the accuracy of the MODIS LAI has not been extensively evaluated at high latitudes because of limited ground measurements and few valid MODIS data points due to inadequate sun-sensor geometry, illumination conditions, and cloud contamination^69,70^. Furthermore, the heterogeneous landscapes of the region at the scale of remote sensing data (from hundreds of meters to a few kilometers) are also a major challenge that must be addressed before the data can be evaluated against ground measurements. According to the spatial representativeness assessment (see Supplementary Note, and Figs. S7 and S8), the landscapes around the flux towers generally have heterogeneity at a level similar to, or smaller than, the tower footprint size (200–300 m) during the early GS and peak GS in the MODIS 1 × 1 pixel window (i.e., 500 × 500 m^2^), but mostly higher than in the 3 × 3 pixel window (Table S6). This indicates that it is desirable to evaluate the MODIS 1 × 1 pixel LAI values against ground measurements, as both MODIS greenup and snowmelt timings agreed more with the ground data at the 1 × 1 pixel window scale than at the 3 × 3 pixel window scale (Figs. S9 and S10). A more thorough evaluation of both MODIS LAI data and ED2 LAI values is required in the coming years with the increase in ground data availability (e.g., National Ecological Observatory Network, NEON, LAI measurements).

### Correlation analysis: the effects of early or delayed snowmelt timing

To analyze the net and lagged effects of early or late snowmelt timing, it is necessary to constrain the contribution of interannual meteorological variation. Therefore, we compared only the years when meteorological conditions were similar, i.e., when the weekly mean GSI value was within one standard deviation of the weekly mean GSI during 2001–2018 (at the US-Hva site, weekly values during 1994–2018); meteorological conditions appeared similar for 8 or 9 years at each study site, except the US-BZF site, where the similarities were found for 10 years. We also limited the effect of greenup timing changes by excluding the years when greenup was earlier or later by one standard deviation of the mean of greenup timings during the study period. For the years satisfying the constraints, a least-squares linear regression was applied between the snowmelt timing change (deviation in snowmelt timing each year from the mean snowmelt timing) and the seasonal deviation (the difference of the seasonal mean from the mean value over the years) of each process from the ED2 results.

To analyze the net and lagged effects of delayed snowmelt, we implemented the ED2 model in two schemes, (1) following the meteorologically-determined phenological index (i.e., the GSI, Eqs. (2) and (2) constraining leaf-out by snowmelt (i.e., the SGSI, Eq. (3)). For the years when unmelted snow delayed greenup, we took the difference between the modeled results (i.e., GSI and SGSI) for each process and applied a least-squares linear regression between the difference of each process and the delayed snowmelt days.

## Supplementary information

Supplementary Info

## Data Availability

European Centre for Medium-Range Weather Forecast Reanalysis v5 (ERA5) is publicly available from https://www.ecmwf.int/en/forecasts/datasets/reanalysis-datasets/era5. North American Regional Reanalysis (NARR) data is available from the NOAA Physical sciences Laboratory via https://psl.noaa.gov/data/gridded/data.narr.monolevel.html. Snow depth data of the National Climatic Data Center (NCDC) stations is available from the NOAA via https://www.ncdc.noaa.gov/cdo-web/search. Landsat surface reflectance data is available from the USGS EarthExplorer via https://earthexplorer.usgs.gov/. MODIS products (snow cover, phenology, and LAI) is available from the NASA Earthdata via https://earthdata.nasa.gov/. Site data (e.g., flux, snow depth, radiation, and soil temperature) is available from the ABOVE with identifier (10.3334/ORNLDAAC/1562), AmeriFlux with identifiers (10.17190/AMF/1418678 and 10.17190/AMF/1246064), Bonanza Creek Long-Term Ecological Research (http://www.lter.uaf.edu/data), Fluxnet with identifiers (10.18140/FLX/1440067 and 10.18140/FLX/1440073), and Korea Polar Data Center (https://kpdc.kopri.re.kr/). Harmonized World Soil Database v1.1 is available from the International Institute for Applied Systems Analysis via http://www.iiasa.ac.at/web/home/research/researchPrograms/water/HWSD.html. Global Soil Organic Carbon Map is available from the Food and Agriculture Organization of the United Nations via http://www.fao.org/soils-portal/data-hub/soil-maps-and-databases/en/. PhenoCam v2.0 data is available from the PhenoCam Network via https://phenocam.sr.unh.edu/webcam/. Ent-GVSD v1.0b is available upon reasonable request from Nancy Kiang.

## Code availability

ED2 source code is available from https://github.com/EDmodel/ED2. ONEFlux source code is available from https://github.com/AmeriFlux/ONEFlux.
