# Supplementary material for: Carbon response of tundra ecosystems to advancing greenup and snowmelt in Alaska
Source: Nat Commun. 2021 Nov 25;12:6879. doi: 10.1038/s41467-021-26876-7 (PMC8617207; doi:10.1038/s41467-021-26876-7)
Supplement: Supplementary file 1 — Supplementary Info [file 41467_2021_26876_MOESM1_ESM.pdf]

# Supplementary Information

## Carbon response of tundra ecosystems to advancing greenup and snowmelt in Alaska

JiHyun Kim[1], Yeonjoo Kim[1]*, Donatella Zona[2,3], Walter Oechel[2,4], Sang-Jong Park[5], Bang-Yong Lee[5], Yonghong Yi[6], Angela Erb[7], and Crystal L. Schaaf[7]

[1]Department of Civil and Environmental Engineering, Yonsei University, Seoul, Republic of Korea

[2]Department of Biology, San Diego State University, San Diego CA, USA

[3]Department of Animal and Plant Science, University of Sheffield, Sheffield, UK

[4]Department of Geography, University of Exeter, UK

[5]Division of Atmospheric Sciences, KOPRI, Incheon, Republic of Korea

[6] Joint Institute for Regional Earth System Science and Engineering, University of California, Los Angeles, CA, USA

[7]School for the Environment, University of Massachusetts Boston, Boston, MA, USA

*Corresponding author. Email: yeonjoo.kim@yonsei.ac.kr (Y.K.)

**Supplementary Note: Spatial representativeness assessment and MODIS greenup and snowmelt timing evaluation**

We assessed the spatial representativeness of the landscape within the tower footprint (diameter of 200-300 m; Fig. S7) for the Moderate resolution Imaging Spectroradiometer (MODIS) gridded spatial scales (500×500 $m^2$ and 1.5×1.5 $km^2$ subsets via 1×1 and 3×3 pixel windows, respectively, Fig. S8) at each site following Román et al. (2009)[1]. First, variogram estimators were obtained from the enhanced vegetation index (EVI) retrieved from 30-m Landsat TM and OLI level-2 surface reflectance (https://earthexplorer.usgs.gov/) for seven flux tower sites (during the snowmelt season, early GS, and peak GS), five National Climatic Data Center (NCDC) stations during snowmelt season, and six PhenoCam sites[2] during the early GS (Fig. S1A, Tables S1 and S3). We then derived the variogram model parameters (*nugget effect*, *sill*, and *range* values) by fitting an isotropic spherical variogram model to the variogram estimators. The *range* value indicates the average patch size of the landscape heterogeneity[1]; therefore, we assumed that the landscape around the tower is likely to be representative of the MODIS in the pixel window (e.g., 1×1 pixel or 3×3 pixels) when the *range* value at the pixel window is smaller than (or close to) the tower footprint size (approximately 250 m)[3].

Fig. S8 and Table S3 show that the *range* values of the flux tower sites are smaller than (or close to) the tower footprint size in the 1×1 pixel windows over the seasons (i.e., snowmelt, early GS, and peak GS) but higher than the tower footprint size in the 3×3 pixel windows at most sites. This indicates that the landscapes around the towers are likely to be representative for the MODIS 1×1 pixel windows but less (or even not) representative for the 3×3 pixel windows. PhenoCam sites, except imcrkridge0 and NEON-D19-HEAL, are spatially representative of the MODIS 1×1 pixel windows during the early GS. Four NCDC stations are likely representative in the 1×1 pixel windows during the snowmelt season, but the USC00503585 station may not be spatially representative in either pixel window given that the *range* values are far above 250 m.

For snowmelt timing validation, we collected ground data, including snow depth measurements at the KOPRI site (available upon request from *S. Park*) and five NCDC stations (https://www.ncdc.noaa.gov/cdo-web/, Table S2), and incoming and outgoing short wavelength radiation data at the US-EML site (https://doi.org/10.17190/AMF/1418678). Snowmelt timing was estimated as the day when the amplitude of the ground data (i.e., snow depth or the ratio of incoming to outgoing short-wavelength radiation) dropped below 10% of the wintertime mean value.

We also calculated the MODIS snowmelt timing when a logistic fit to the MODIS snow cover (MOD10A1.V006, quality flags of good and best) passed 0.1 each year and evaluated it against ground-based estimates (Fig. S9). Our results show that the MODIS snowmelt timings agree well with those from ground data where the site was spatially representative during the snowmelt season at the MODIS scale (Fig. S9A, $p < 0.001$). We also found that the agreement is higher at a more representative scale (i.e., higher $r_{adj}^2$ at the 1×1 pixel window, Fig. S9B). Poor agreement was also found at the USC00503585 station (which was spatially not representative in either pixel window, Fig. S9C).

PhenoCam dataset v2.0[2] (https://phenocam.sr.unh.edu/webcam/, Table S2) was used to validate the MODIS greenup timing (MCD12Q2.V006, quality flag of best). In the dataset, we used the 10% amplitude threshold date during "greenness rising", which was shown to have the smallest bias against the MODIS greenup (8 ± 21 days across 12 land cover types; dataset v1.0[4]). We confirmed that the MODIS greenup generally agrees well with the PhenoCam data in the MODIS 1×1 pixel windows when only including the spatially representative sites (root-mean-squared value, $RMS = 10.0$ days, Fig. S10) and worse in the 3×3 pixel windows (average value of the nine pixels, $RMS = 17.6$ days). However, it should be noted that the number of data points is still very limited ($N \leq 11$); therefore, further analysis with longer data periods including more sites should be performed.

Our results imply that it is critical to consider the implication of spatial representativeness on remote sensing-based timing estimates[5,6]. Given that the flux tower sites are mostly representative in the MODIS 1×1 pixel windows during both the snowmelt season and early GS (Table S3), MODIS snowmelt and greenup timings were applied for further analysis in this study. Due to the low availability of leaf area index (LAI) measurements at the tower sites, MODIS LAI data (MCD15A3H.V006) evaluation in terms of spatial representativeness was not performed in this study. However, given that the landscapes around the towers are mostly representative of the MODIS 1×1 pixel windows during the early and peak GS, there would be less uncertainty in the MODIS LAI data resulting from landscape heterogeneity; therefore, we used the MODIS LAI values to calibrate and evaluate ED2.

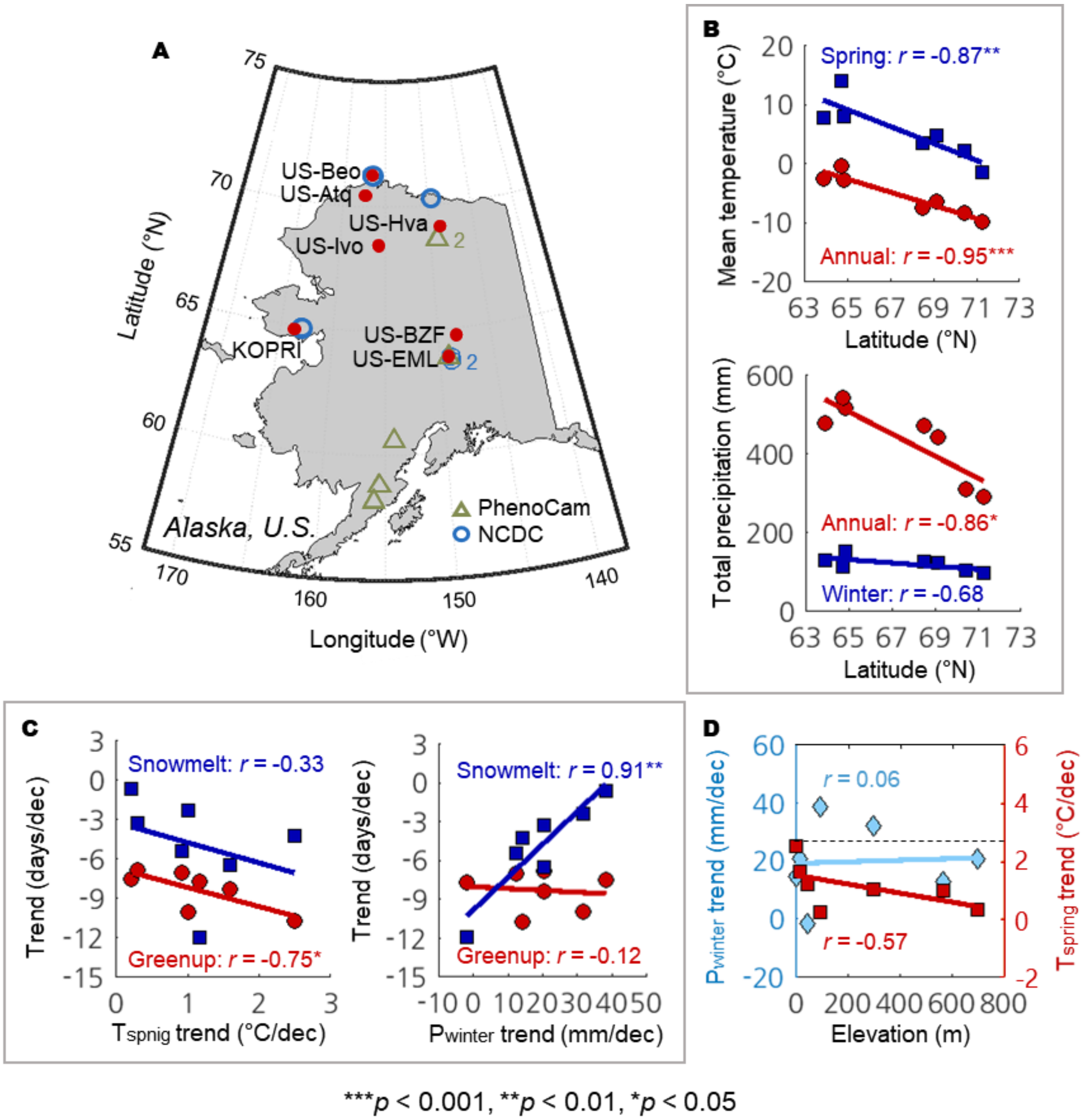


**Fig. S1. Study site location, climatic gradients, and correlations between trends.** (A) Flux tower sites (red dots), PhenoCam sites (green triangles), and NCDC stations (blue circles) across Alaska in the United States (Tables S1 and S3, a number is provided when the sites/stations are too close to be separated in the map). Detailed description is available at Methods-Study sites. (B) Latitudinal gradients in temperature (annual and spring between Apr. and June) and precipitation (annual and winter from Jan. to May). (C) Correlation of greenup and snowmelt timings with the spring mean temperature ($T_{spring}$) trend and winter precipitation ($P_{winter}$) trend. (D) Correlations of $T_{spring}$ and $P_{winter}$ with elevation.

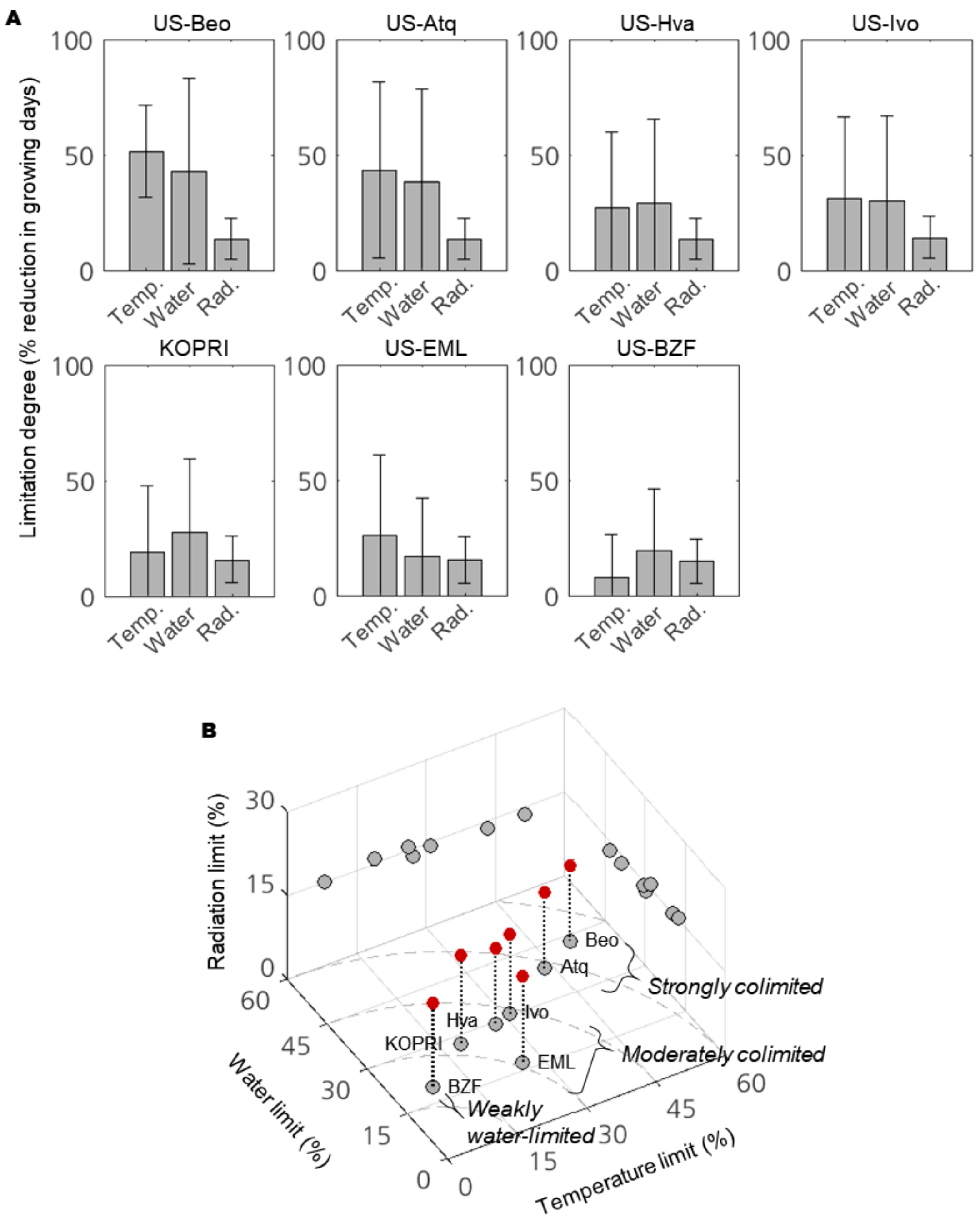


**Fig. S2. Climatic limit estimation.** (A) Climatic limits (% reduction in growing days) by temperature, water, and radiation at each site (see Methods-Study sites). Bars and error bars indicate the mean and standard deviation values, respectively, during the growing season (May - Oct.) from 2001 to 2018. (B) Study sites are classified as "strongly colimited", "moderately colimited", and "weakly water-limited" sites depending on the climatic limits.

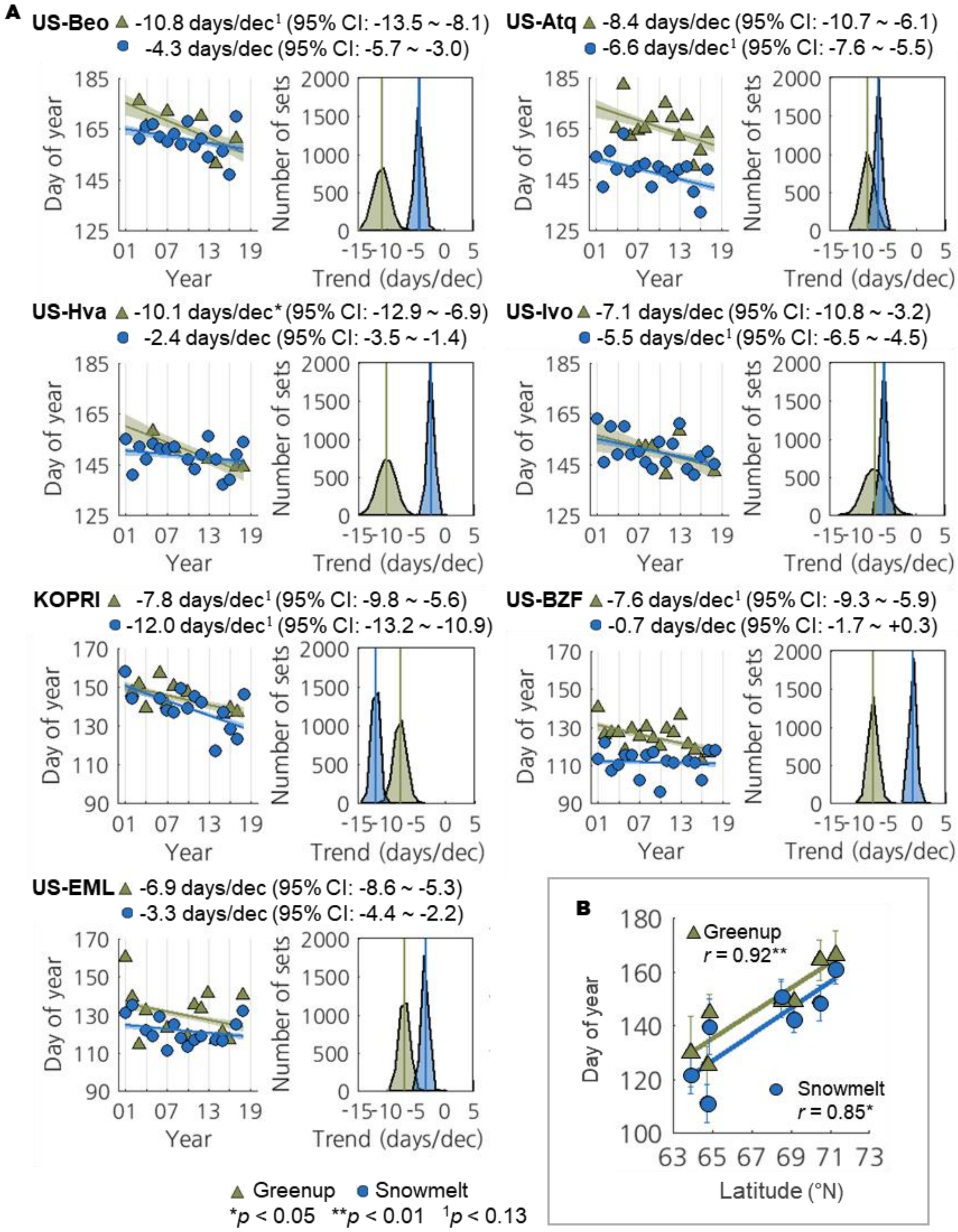


**Fig. S3. Greenup and snowmelt timings and long-term trends.** (A) Greenup timing (green triangles, MCD12Q2.V006) and snowmelt timing (blue circles, derived from MODIS snow cover, MOD10A1.V006) for eighteen years (2001–2018) at each study site and their long-term trends (green line for the greenup trend and blue line for the snowmelt timing trend, determined by Spearman's rho and Mann-Kendall tests). The shaded area represents a 95% confidence interval of the trend lines of 3,000 bootstrap-sampled timing sets (see Methods-MODIS). The distribution plot shows the trends of the 3,000 timing sets at each site. (B) Latitudinal gradients in both snowmelt and greenup timings (2001–2018 means in symbols and one std. dev. in vertical lines at each site).

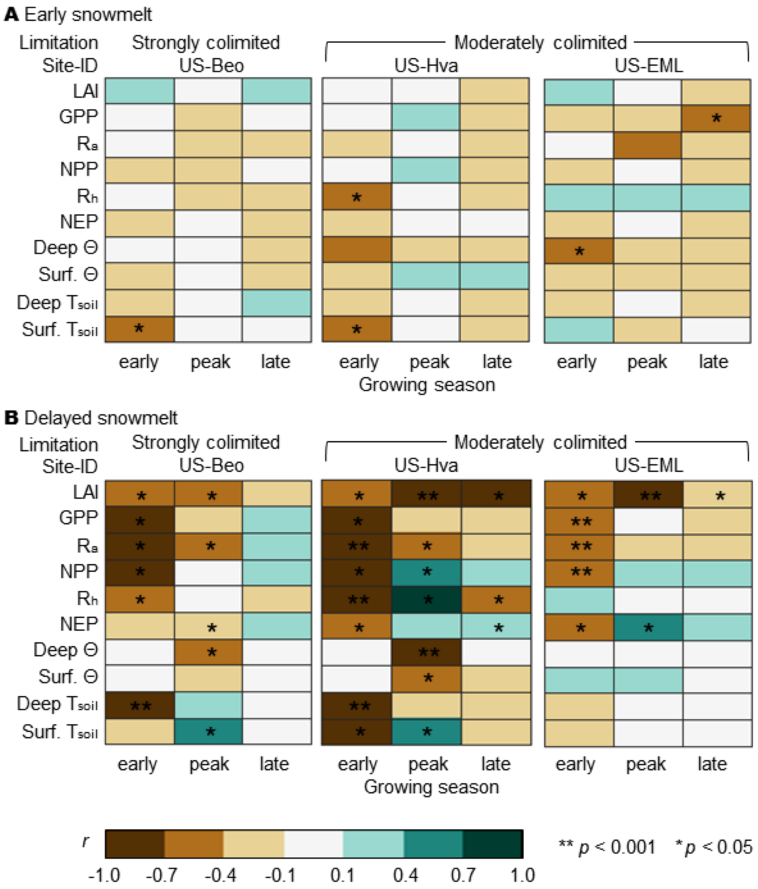


**Fig. S4. Seasonal effects of early or delayed snowmelt timing.** Pearson correlation (*r*) between each ecosystem process and (A) early snowmelt and (B) delayed snowmelt during each growing season at the strongly temperature and water-colimited site (US-Beo) and the moderately colimited sites (US-Hva and US-EML) under similar meteorological conditions (based on weekly GSI values) and greenup timings. Other sites, including the weakly water-limited site (US-BZF), the strongly colimited site (US-Atq), and the moderately colimited sites (US-Ivo and KOPRI), are presented in Fig. 4.

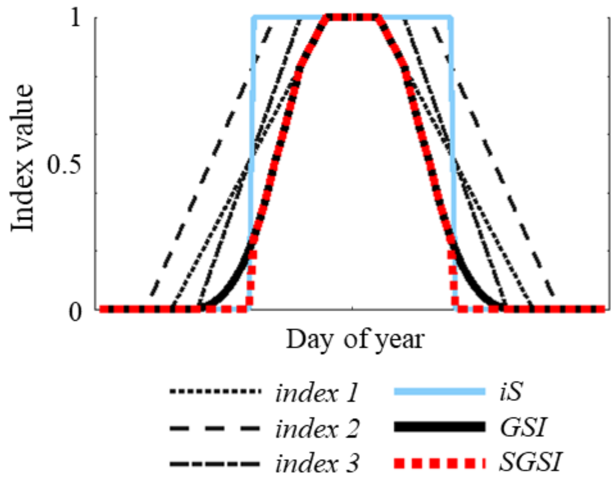


**Fig. S5. Schematic illustration of the growing season index (GSI) and snowmelt-growing season index (SGSI).** The growing season index (GSI, Eq. 1, black solid line) is a product of three indices (daylength-, vapor pressure deficit-, and growing-degree-days-based indices, i.e., indices 1–3 indicated by dotted-, dashed- and dot-dashed lines), and the snowmelt-growing season index (SGSI, red dotted line, Eq. 2) is the product of the GSI and a snowmelt timing index (*iS*, blue sold line).

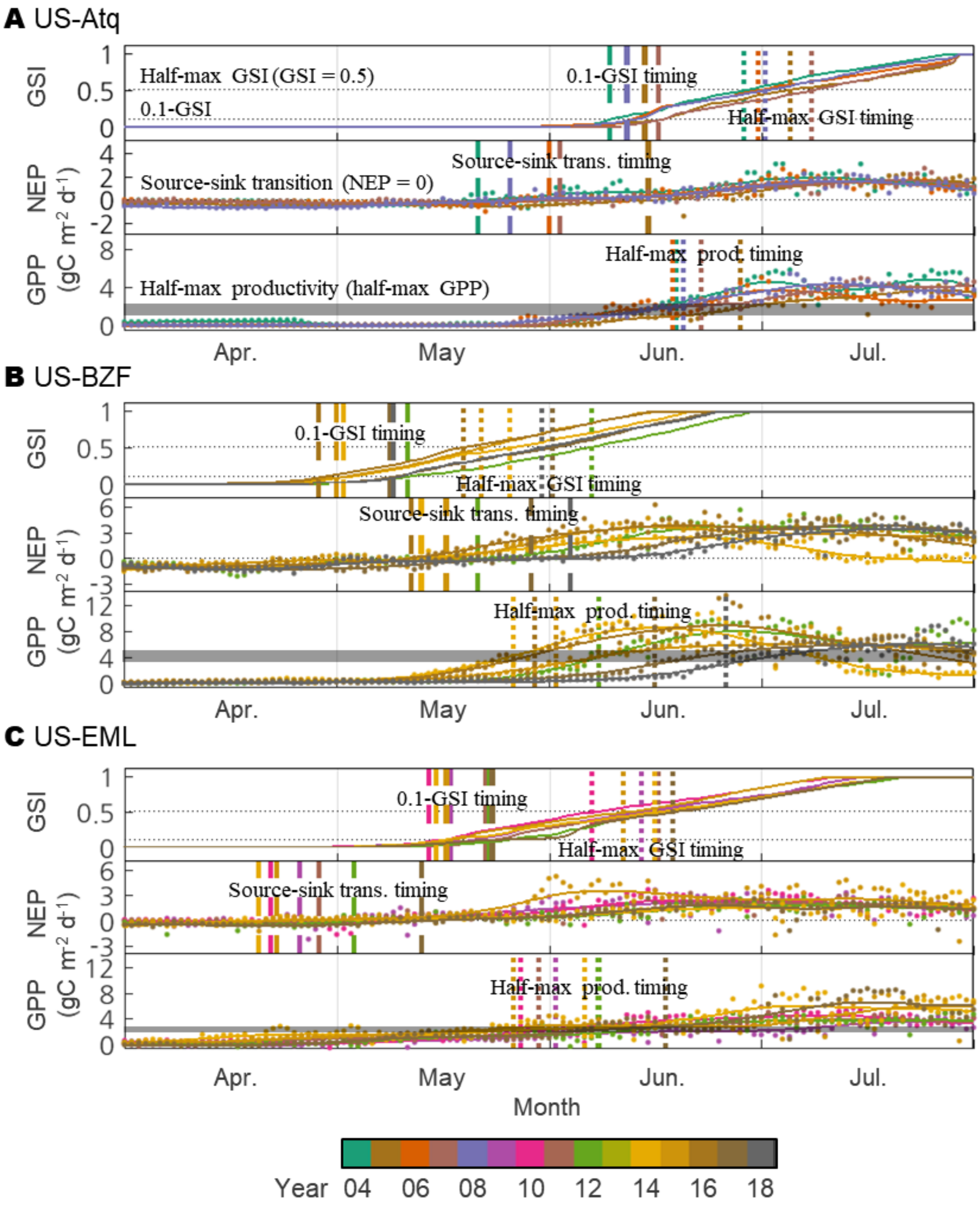


**Fig. S6. Threshold timing estimations at (A) the US-Atq, (B) US-BZF, and (C) US-EML sites.** Half-max growing seasonal index (GSI) and 0.1 GSI timings (vertical dotted lines and dashed lines, respectively) when the daily GSI passes 0.5 and 0.1 values, respectively. Source-sink transition timing when the smoothing spline of daily NEP passes zero, and half-max productivity timing when the smoothing spline of daily GPP passes the half-max value of the year (see Methods-Case study).

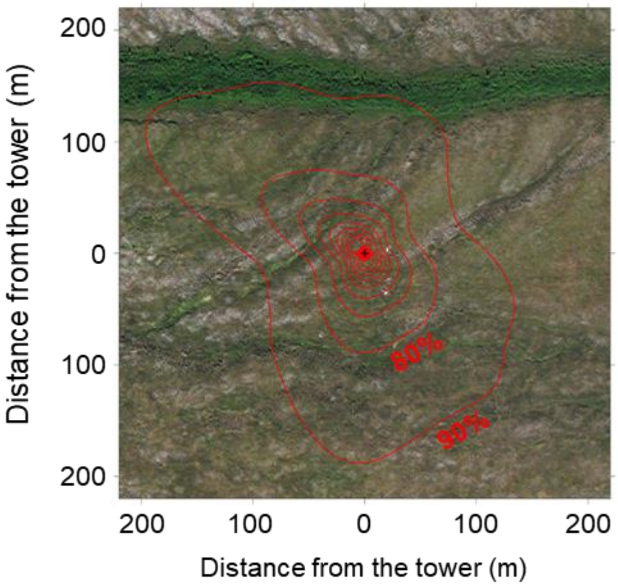


**Fig. S7. Footprint climatology of the KOPRI tower in 2013.** The extent of the source areas (from 10% to 90% with the 10% interval in the red contour) of the KOPRI tower (black cross) in 2013 (by *S. Park*)**.**

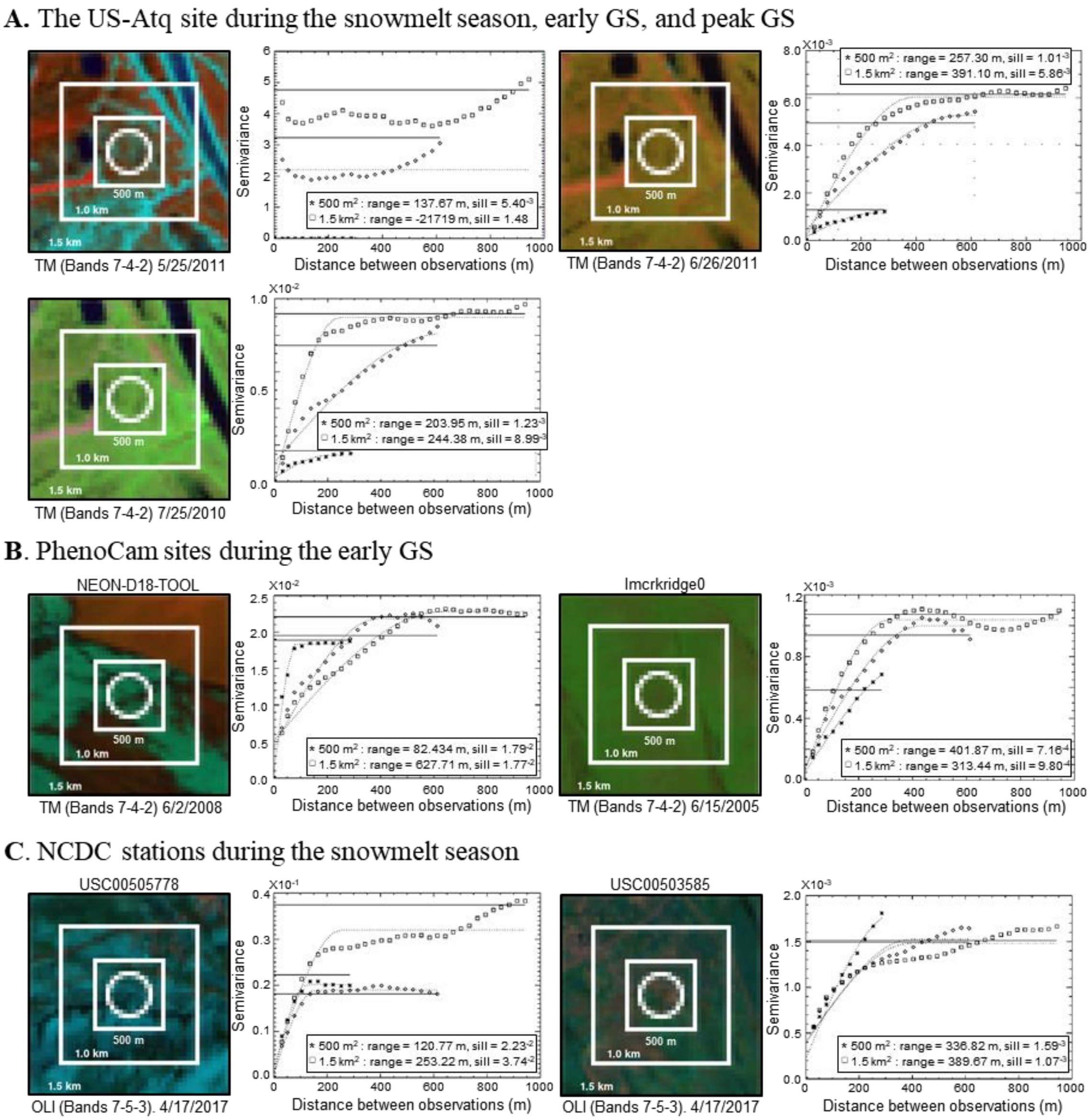


**Fig. S8. Schematic examples of spatial representativeness assessment.** Surface reflectance composite (Landsat TM Bands 7-4-2; OLI Bands 7-5-3) centered on (A) a flux tower, US-Atq, during the snowmelt season, early GS and peak GS, (B) two PhenoCam sites during the early GS, and (C) two NCDC during the snowmelt season with 500×500 $m^2$, 1.0×1.0 $km^2$, and 1.5×1.5 $km^2$ boundaries (squares) and the region of interest (circle, diameter of 250 m). Variogram estimators were obtained from the enhanced vegetation index (EVI) retrieved from Landsat scenes for each subset (asterisks, diamonds, and squares for 500×500 $m^2$, 1.0×1.0 $km^2$, and 1.5×1.5 $km^2$ subsets, respectively), spherical models were fitted (dotted curves), and then variance values were found (horizontal solid lines). A detailed description is available in the Supplementary note.

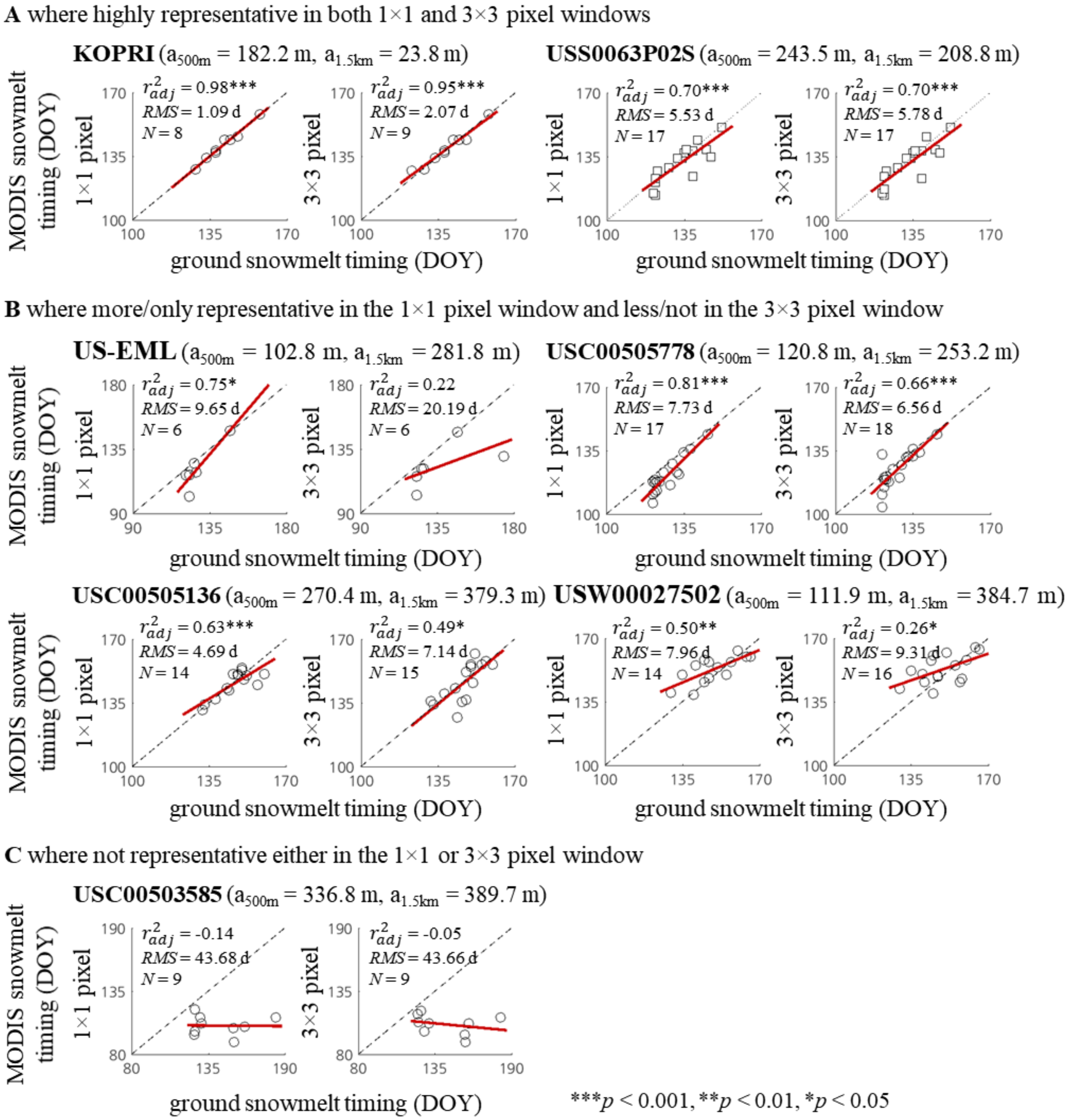


**Fig. S9. Evaluation of MODIS snowmelt timing.** MODIS snowmelt timing estimated from the MODIS snow cover (MOD10A1.V006) in the 1×1 and 3×3 pixel windows compared to the ground snowmelt timing with the *range* value (a) from the spatial representativeness assessment at NCDC stations and flux towers (see Supplementary Note).

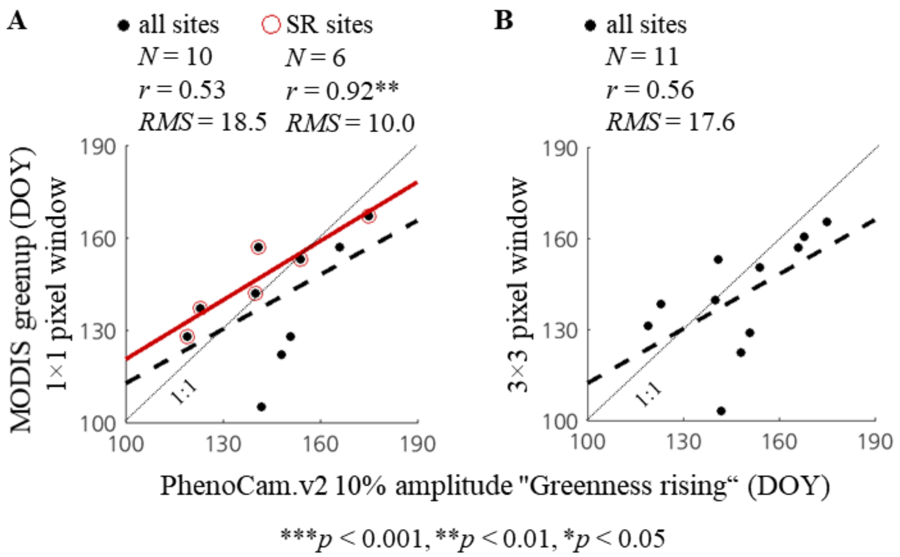


**Fig. S10. Evaluation of MODIS greenup timing.** MODIS greenup timing (MCD12Q2.V006) in the 1×1 and 3×3 pixel windows (average value), respectively, compared to the greenness rising timing derived from PhenoCam dataset v2.0[2]. A detailed description of the spatial representativeness (SR) assessment is available in the Supplementary note.

**Table S1. List of study sites.** Basic information for the flux tower study sites

| Site ID[a] | Coordinates[a] | Elevation[a] | Meteorological conditions[b] | Plant functional type[c] | Permafrost depth |
|---|---|---|---|---|---|
| **US-Beo** | 71.2810 °N<br>156.6123 °W | 1 m | -10.09 ± 0.96 °C<br>287.24 ± 50.75 mm<br>1218.9 ± 22.6 $kW/m^2$ | Arctic C3 grass | 0.33 m[7] |
| **US-Atq** | 70.4696 °N<br>157.4089 °W | 15 m | -8.48 ± 0.81 °C<br>308.99 ± 56.87 mm<br>1120.4 ± 20.6 $kW/m^2$ | Arctic C3 grass,<br>Cold-adapted shrub | 0.50 m[7] |
| **US-Hva** | 69.1423 °N<br>148.8412 °W | 298 m | -6.57 ± 0.89 °C<br>440.75 ± 123.12 mm<br>1262.3 ± 24.9 $kW/m^2$ | Cold-adapted shrub | 0.46 m[8] |
| **US-Ivo** | 68.4865 °N<br>155.7503 °W | 568 m | -7.59 ± 0.90 °C<br>469.65 ± 68.99 mm<br>1127.9 ± 14.1 $kW/m^2$ | Cold-adapted shrub | 0.56 m[8] |
| **KOPRI** | 64.8433 °N<br>163.7053 °W | 45 m | -3.01 ± 1.27 °C<br>515.85 ± 67.35 mm<br>1177.2 ± 21.9 $kW/m^2$ | Arctic C3 grass,<br>Cold-adapted shrub | 0.60 m[9] |
| **US-BZF** | 64.7037 °N<br>148.3133 °W | 95 m | -0.55 ± 0.78 °C<br>540.12 ± 115.34 mm<br>1215.8 ± 17.5 $kW/m^2$ | Cold-adapted shrub | 1.0 m[8] |
| **US-EML** | 63.8784 °N<br>149.2536 °W | 700 m | -2.64 ± 1.04 °C<br>474.07 ± 73.64 mm<br>1190.5 ± 18.2 $kW/m^2$ | Arctic C3 grass,<br>Cold-adapted shrub | 0.60 m[10] |

[a]AmeriFlux (https://ameriflux.lbl.gov/) and Korea Polar Research Institute (KOPRI; https://kpdc.kopri.re.kr/)
[b]Annual mean temperature, total precipitation, and total radiation (mean ± std. dev. during 2001–2018) from the 3-hourly North American Regional Reanalysis (NARR) dataset[11] (https://psl.noaa.gov/data/gridded/data.narr.monolevel.html)
[c]Ent-GVSD v1.0b (available upon reasonable request from Nancy Kiang)

**Table S2. Summary of the stepwise multiple regression model** (Eq. 1) between the NEE and meteorological variables (temperature, VPD, and PAR) during the GS 2001-2018.

| | | **Variable** | | | | | | | |
|---|---|---|---|---|---|---|---|---|---|
| | | **(Intercept)** | **TEMP** | **PAR** | **VPD** | **TEMP:PAR** | **TEMP:VPD** | **PAR:VPD** | **TEMP:PAR:VPD** |
| **US-Beo** $R^2$ = 0.518 | Coefficient | 1.05E+00 | 3.14E-02 | -3.46E-03 | -7.85E-03 | -3.15E-04 | - | 1.90E-05 | - |
| | Std. Error | 1.79E-01 | 1.71E-02 | 5.96E-04 | 1.81E-03 | 6.35E-05 | - | 4.93E-06 | - |
| | *P*-value | *** | *** | *** | ** | *** | - | *** | - |
| **US-Atq** $R^2$ = 0.387 | Coefficient | 5.07E-01 | 2.59E-02 | -2.49E-03 | -5.94E-03 | -3.26E-04 | 2.13E-04 | 1.66E-05 | -3.70E-07 |
| | Std. Error | 7.90E-02 | 7.94E-03 | 2.65E-04 | 1.24E-03 | 2.79E-05 | 8.98E-05 | 3.06E-06 | 1.99E-07 |
| | *P*-value | *** | *** | *** | *** | *** | * | *** | |
| **US-Hva** $R^2$ = 0.644 | Coefficient | 3.83E-01 | -4.55E-02 | -9.25E-04 | 1.66E-03 | -1.22E-04 | -7.36E-05 | - | - |
| | Std. Error | 2.13E-01 | 2.41E-02 | 5.55E-04 | 7.08E-04 | 4.37E-05 | 4.24E-05 | - | - |
| | *P*-value | | * | | * | ** | | - | - |
| **US-Ivo** $R^2$ = 0.627 | Coefficient | 1.04E+00 | 3.87E-02 | -2.99E-03 | -1.48E-03 | -4.45E-04 | -9.48E-05 | 9.80E-06 | - |
| | Std. Error | 6.74E-02 | 7.03E-03 | 2.58E-04 | 5.23E-04 | 3.32E-05 | 3.05E-05 | 1.06E-06 | - |
| | *P*-value | *** | ** | ** | ** | *** | ** | *** | - |
| **KOPRI** $R^2$ = 0.680 | Coefficient | 6.82E+00 | -4.58E-01 | -1.09E-02 | -5.61E-03 | - | 3.71E-04 | - | - |
| | Std. Error | 2.99E+00 | 1.78E-01 | 1.27E-03 | 2.74E-03 | - | 1.52E-04 | - | - |
| | *P*-value | * | * | ** | * | - | * | - | - |
| **US-BZF** $R^2$ = 0.482 | Coefficient | 2.68E-01 | -3.55E-02 | -1.56E-03 | 2.32E-03 | -5.41E-04 | -1.41E-04 | 6.68E-06 | - |
| | Std. Error | 1.18E-01 | 1.30E-02 | 7.72E-04 | 5.47E-04 | 6.34E-05 | 2.96E-05 | 7.54E-07 | - |
| | *P*-value | * | * | * | *** | ** | *** | *** | - |
| **US-EML** $R^2$ = 0.547 | Coefficient | 5.89E-01 | -1.79E-02 | -1.37E-03 | 8.05E-04 | -4.24E-04 | -5.44E-05 | -3.20E-07 | 2.09E-07 |
| | Std. Error | 8.21E-02 | 8.46E-03 | 3.86E-04 | 2.72E-04 | 3.22E-05 | 1.69E-05 | 8.02E-07 | 4.11E-08 |
| | *P*-value | *** | * | ** | ** | *** | ** | | *** |

***$P < 0.001$ **$P < 0.01$ *$P < 0.05$ $P > 0.05$

**Table S3. List of PhenoCam sites and NCDC stations.** Coordinates and data period of the PhenoCam sites and NCDC stations where the spatial representativeness assessment and the evaluation of MODIS greenup and snowmelt timings were performed (see Supplementary Note and Fig. S1A).

| | | Coordinate | Data period (year) |
|---|---|---|---|
| **PhenoCam site**[a] | **contactcreek** | 58.2076 °N, 155.9225 °W | 2012 |
| | **coville** | 58.8025 °N, 155.5629 °W | 2011–2013 |
| | **snipelake** | 60.6103 °N, 154.3199 °W | 2011, 2012 |
| | **imcrkridge0** | 68.6068 °N, 149.2958 °W | 2013, 2017, 2018 |
| | **NEON-D18-TOOL** | 68.6611 °N, 149.3705 °W | 2017, 2018 |
| | **NEON-D19-HEAL** | 63.8757 °N, 149.2133 °W | 2016–2018 |
| **NCDC station**[b] | **USC00505778** | 63.7175 °N, 148.9690 °W | 2001–2018 |
| | **USW00027502** | 71.2834 °N, 156.7815 °W | 2001–2018 |
| | **USS0063P02S** | 64.9900 °N, 163.1000 °W | 2001–2018 |
| | **USC00505136** | 70.3289 °N, 149.6110 °W | 2001 – 2018 |
| | **USC00503585** | 63.8674 °N, 148.9940 °W | 2001–2016 |

[a]PhenoCam Network (https://phenocam.sr.unh.edu/webcam/)
[b]National Climatic Data Center (NCDC) stations (https://www.ncdc.noaa.gov/cdo-web/search)

**Table S4. Summary of key ED2 variables.** The prior distribution of the key variables that were calibrated in this study (distribution type and [parameters a, b])

| **Variable** | **Plant functional type** | | |
|---|---|---|---|
| | Graminoid | Deciduous shrub | Evergreen shrub |
| Specific leaf area ($m^2$ $kgC^{-1}$)[12–16] | Uniform [4.4, 23.2] | Uniform [7.8, 14.9] | Uniform [3.6, 15.0] |
| Carboxylation max rate (μmol $m^{-2}$ $s^{-1}$)[17] | Weibull [3.55, 24.7] | | |
| Photosynthetic min temperature (°C)[18] | Normal [-3, 2] | | |
| Growth respiration factor[18] | Beta [4.06, 7.2] | Beta [2.63, 6.52] | Beta [4.06, 7.2] |
| Fine root allocation | Log-Normal [0.81, 0.84] | | |
| Allometric parameters[a] | | | |
| DBH-height intercept (b1Ht) | 0.4778 | 3.0 | 1.0 |
| DBH-height exponent (b2Ht) | -0.75 | -0.4 | -0.7 |
| DBH-leaf intercept (b1Bl) | 0.045 | 0.08 | 0.013 |
| DBH-leaf exponent (b2Bl) | 1.68 | 1.0 | 1.75 |
| DBH-stem intercept (b1Bs) | 0.05 | 0.00001 | 0.0265 |
| DBH-stem exponent (b2Bs) | 2.15 | 1.0 | 2.95 |

[a]derived by Eric Larson using[19,20]

**Table S5. Summary of ED2 calibration and validation.** The dataset, period, and statistical measures ($r^2$ and root-mean-square-error, RMSE) for each variable used for calibration at the US-Atq site (c) and evaluation at the seven study sites

| Site ID | **Net ecosystem productivity (NEP; 30-min)** Dataset, Period $r^2$, RMSE (μmol $m^{-2}$ $s^{-1}$) | **Ecosystem respiration (Reco; 30-min)** Dataset, Period $r^2$, RMSE (μmol $m^{-2}$ $s^{-1}$) | **Soil temperature (Daily mean)** Dataset, Period $r^2$, RMSE (°C) | **Leaf area index (LAI; 4-day)**[a] $r^2$, RMSE ($m^2$ $m^{-2}$) | **Snowmelt timing**[b] $r^2$, RMSE (days) | **Greenup timing**[c] $r^2$, RMSE (days) |
|---|---|---|---|---|---|---|
| US-Beo | ABoVE[d] 2015 – 16 0.54, 1.18 | N/A | ABoVE[d] 2015 – 16 0.82, 3.61 | 0.76, 0.14 | 0.49, 4.88 | 0.51, 5.58 |
| US-Atq | FLUXNET2015[e,f,21] (c) 2004 – 06, 0.66, 1.21 2007 – 08, 0.64, 1.17 | FLUXNET2015[e,21] 2004 – 08 0.58, 1.23 | FLUXNET2015[e,f,21] 2003 – 08 0.92, 2.89 | (c) 2003 – 10, 0.83, 0.16 2011 – 18, 0.86, 0.16 | 0.61, 4.67 | 0.46, 5.52 |
| US-Hva | AmeriFlux[g,h,22] 1994 – 95 0.54, 1.89 | N/A | AmeriFlux[g,22] 1995 0.47, 3.95 | 0.87, 0.14 | 0.58, 4.04 | 0.56, 4.94 |
| US-Ivo | FLUXNET2015[e,f,23] 2004 – 07 0.59, 1.6 | FLUXNET2015[e,23] 2004 – 07 0.62, 0.95 | FLUXNET2015[e,f,23] 2004 – 07 0.92, 3.05 | 0.90, 0.13 | 0.49, 4.35 | 0.53, 6.40 |
| KOP RI | KPDC[f,i] 2014 – 15 0.59, 2.57 | KPDC[j] 2014 – 15 0.47, 0.76 | N/A | 0.74, 0.28 | 0.64, 3.97 | 0.58, 4.49 |
| US-BZF | Bonanza LTER[j] 2014 – 18 0.51, 2.16 | N/A | Bonanza LTER[j] 2014 – 18 0.67, 4.42 | 0.79, 0.30 | 0.79, 4.04 | 0.54, 3.92 |
| US-EML | AmeriFlux[g,h,24] 2008 – 17 0.66, 2.08 | AmeriFlux[g,24] 2008 – 17 0.59, 0.89 | AmeriFlux[g,24] 2008 – 15 0.78, 3.85 | 0.87, 0.26 | 0.62, 4.13 | 0.49, 5.37 |

[a]MODIS LAI (MCD15A3H.V006[25] from NASA Earthdata; https://earthdata.nasa.gov/) between 2003 and 2018 with quality flags of good and no clouds.

[b]MODIS snow cover (MOD10A1.V006[26] from NASA Earthdata)-based snowmelt timing (when the logistic fit to the snow cover passes 0.1) between 2001 and 2018

[c]MODIS phenology greenup timing (MCD12Q2.V006[27] from NASA Earthdata) between 2001 and 2018. Quality flag of best

[d]Arctic-Boreal Vulnerability Experiment (https://doi.org/10.3334/ORNLDAAC/1562)[28]

[e]FLUXNET2015 (https://fluxnet.fluxdata.org/data/fluxnet2015-dataset/)

[f]Quality flags: measured or good

[g]AmeriFlux (https://ameriflux.lbl.gov/)

[h]Flux points (half-hourly) were removed when u* < 0.25 m $s^{-1}$, and then gaps (< 2 hours) were linearly filled[29]

[i]Korea Polar Data Center (https://kpdc.kopri.re.kr/)

[j]Bonanza Creek Long-Term Ecological Research (http://www.lter.uaf.edu/data)[30,31]

**Table S6. Summary of the spatial representativeness assessment.** The variogram parameters (*range* and *sill* values, see Supplementary Note) at the flux tower sites (a) and PhenoCam sites and NCDC stations (b) for the 500×500 $m^2$ and 1.5×1.5 $km^2$ subsets (i.e., MODIS 1×1 and 3×3 pixel windows, respectively), and Landsat scene information (instrument and acquisition date) used to derive variogram estimators.

(a) Flux tower sites

| | **Snowmelt season** | **Early GS** | **Peak GS** |
|---|---|---|---|
| **US-Beo** | 500 $m^2$: 90.2, $1.5^{-5}$<br>1.5 $km^2$: 277.4, $2.7^{-5}$<br>TM. 6/14/1986 | 500 $m^2$: 161.9, $1.1^{-3}$<br>1.5 $km^2$: 605.9, $3.7^{-3}$<br>TM. 6/19/2011 | 500 $m^2$: 137.8, $9.8^{-4}$<br>1.5 $km^2$: 942.2, $2.9^{-3}$<br>TM. 7/25/2010 |
| **US-Atq** | 500 $m^2$: 137.7, $5.4^{-3}$<br>1.5 $km^2$: -21719, 1.5<br>TM. 5/25/2011 | 500 $m^2$: 257.3, $1.0^{-3}$<br>1.5 $km^2$: 391.1, $5.9^{-3}$<br>TM. 6/26/2011 | 500 $m^2$: 203.9, $1.2^{-3}$<br>1.5 $km^2$: 244.4, $8.9^{-3}$<br>TM. 7/25/2010 |
| **US-Hva** | 500 $m^2$: 98.9, $5.3^{-3}$<br>1.5 $km^2$: -1889, $5.7^{2}$<br>OLI. 5/24/2017 | 500 $m^2$: 251.7, $2.3^{-3}$<br>1.5 $km^2$: 1403.7, $1.8^{-2}$<br>OLI. 6/10/2020 | 500 $m^2$: 219.2, $1.6^{-3}$<br>1.5 $km^2$: 1238.6, $1.7^{-2}$<br>OLI. 7/17/2019 |
| **US-Ivo** | 500 $m^2$: 114.4, $1.5^{-1}$<br>1.5 $km^2$: 172.4, 15.9<br>TM. 5/14/2018 | 500 $m^2$: 211.6, $4.6^{-3}$<br>1.5 $km^2$: 218.2, $3.9^{-3}$<br>TM. 6/1/2018 | 500 $m^2$: 262.5, $6.1^{-4}$<br>1.5 $km^2$: 366.1, $1.3^{-3}$<br>TM. 7/25/2015 |
| **KOPRI** | 500 $m^2$: 182.2, $3.9^{-2}$<br>1.5 $km^2$: 23.8, $6.5^{-2}$<br>TM. 5/6/2016 | 500 $m^2$: 102.3, $5.9^{-4}$<br>1.5 $km^2$: 1026.9, $1.5^{-3}$<br>OLI. 6/21/2015 | 500 $m^2$: 118.4, $1.1^{-3}$<br>1.5 $km^2$: 326.2, $1.6^{-3}$<br>TM. 8/16/2009 |
| **US-BZF** | 500 $m^2$: 259.2, $2.3^{-3}$<br>1.5 $km^2$: 989.8, $4.7^{-3}$<br>OLI. 4/20/2009 | 500 $m^2$: 216.6, $2.7^{-3}$<br>1.5 $km^2$: 1051.2, $1.1^{-2}$<br>OLI. 6/25/2013 | 500 $m^2$: 164.1, $1.5^{-3}$<br>1.5 $km^2$: 1056.9, $8.1^{-3}$<br>TM. 8/23/2008 |
| **US-EML** | 500 $m^2$: 102.8, $6.4^{-4}$<br>1.5 $km^2$: 281.8, $5.4^{-3}$<br>TM. 5/12/2011 | 500 $m^2$: 225.9, $4.7^{-4}$<br>1.5 $km^2$: 288.9, $2.4^{-3}$<br>TM. 6/17/2016 | 500 $m^2$: 113.4, $3.6^{-4}$<br>1.5 $km^2$: 274.8, $1.4^{-3}$<br>TM. 8/23/2008 |

(b) PhenoCam sites and NCDC stations

| | | |
|---|---|---|
| **PhenoCam site during the early GS** | **contactcreek** | 500 $m^2$: 137.2, $3.6^{-3}$, 1.5 $km^2$: 484.1, $6.1^{-3}$<br>TM. 7/7/2009 |
| | **coville** | 500 $m^2$: 152.9, $4.8^{-3}$, 1.5 $km^2$: 403.7, $8.3^{-3}$<br>TM. 7/7/2009 |
| | **snipelake** | 500 $m^2$: 96.2, $1.3^{-3}$, 1.5 $km^2$: 480.4, $4.2^{-3}$<br>TM. 6/4/2006 |
| | **imcrkridge0** | 500 $m^2$: 401.8, $7.2^{-4}$, 1.5 $km^2$: 313.4, $9.8^{-4}$<br>TM. 6/15/2005 |
| | **NEON-D18-TOOL** | 500 $m^2$: 82.4, $1.8^{-2}$, 1.5 $km^2$: 627.7, $1.8^{-2}$<br>TM. 6/2/2008 |
| | **NEON-D19-HEAL** | 500 $m^2$: 297.7, $6.4^{-4}$, 1.5 $km^2$: 310.5, $8.1^{-4}$<br>OLI. 6/25/2013 |
| **NCDC station during the snowmelt season** | **USC00505778** | 500 $m^2$: 120.8, $2.2^{-2}$, 1.5 $km^2$: 253.2, $3.7^{-2}$<br>OLI. 4/17/2017 |
| | **USW00027502** | 500 $m^2$: 111.9, $4.4^{-3}$, 1.5 $km^2$: 384.7, $4.7^{-3}$<br>TM. 5/25/2011 |
| | **USS0063P02S** | 500 $m^2$: 2435.0, $7.5^{-3}$, 1.5 $km^2$: 208.8, $1.1^{-2}$<br>OLI. 5/4/2015 |
| | **USC00505136** | 500 $m^2$: 270.4, $8.1^{-3}$, 1.5 $km^2$: 379.2, $1.0^{-2}$<br>TM. 6/6/2010 |
| | **USC00503585** | 500 $m^2$: 336.8, $1.6^{-3}$, 1.5 $km^2$: 389.7, $1.1^{-3}$<br>OLI. 4/17/2017 |

## Supplementary References


1. Román, M. O. *et al.* The MODIS (Collection V005) BRDF/albedo product: Assessment of spatial representativeness over forested landscapes. *Remote Sens. Environ.* **113**, 2476–2498 (2009).
2. Seyednasrollah, B. *et al.* Tracking vegetation phenology across diverse biomes using Version 2.0 of the PhenoCam Dataset. *Sci. data* **6**, 222 (2019).
3. Kim, J., Hwang, T., Schaaf, C. L., Kljun, N. & Munger, J. W. Seasonal variation of source contributions to eddy-covariance CO 2 measurements in a mixed hardwood-conifer forest. *Agric. For. Meteorol.* **253**–**254**, 71–83 (2018).
4. Richardson, A. D., Hufkens, K., Milliman, T. & Frolking, S. Intercomparison of phenological transition dates derived from the PhenoCam Dataset V1.0 and MODIS satellite remote sensing. *Sci. Rep.* **8**, 5679 (2018).
5. Klosterman, S. T. *et al.* Evaluating remote sensing of deciduous forest phenology at multiple spatial scales using PhenoCam imagery. *Biogeosciences* **11**, 4305–4320 (2014).
6. Liu, Y. *et al.* Using data from Landsat, MODIS, VIIRS and PhenoCams to monitor the phenology of California oak/grass savanna and open grassland across spatial scales. *Agric. For. Meteorol.* **237**–**238**, 311–325 (2017).
7. Zona, D. *et al.* Cold season emissions dominate the Arctic tundra methane budget. *Proc. Natl. Acad. Sci.* **113**, 40–45 (2016).
8. Yi, Y. *et al.* Characterizing permafrost active layer dynamics and sensitivity to landscape spatial heterogeneity in Alaska. *Cryosph.* **12**, 145–161 (2018).
9. Kim, Y. *et al.* Constraint of soil moisture on CO2 efflux from tundra lichen, moss, and tussock in Council, Alaska, using a hierarchical Bayesian model. *Biogeosciences* **11**, 5567–5579 (2014).
10. Salmon, V. G. *et al.* Nitrogen availability increases in a tundra ecosystem during five years of experimental permafrost thaw. *Glob. Chang. Biol.* **22**, 1927–1941 (2016).
11. Mesinger, F. *et al.* North American Regional Reanalysis. *Bull. Am. Meteorol. Soc.* **87**, 343–360 (2006).
12. Reich, P. B. *et al.* Generality of leaf trait relationships: A test across six biomes. *Ecology* **80**, 1955–1969 (1999).
13. Matthes-Sears, U., Matthes-Sears, W. C., Hastings, S. J. & Oechel, W. C. The Effects of Topography and Nutrient Status on The Biomass, Vegetative Characteristics, and Gas Exchange of Two Deciduous Shrubs on An Arctic Tundra Slope. *Arct. Alp. Res.* **20**, 342–351 (1988).
14. Shaver, G. R. *et al.* Species composition interacts with fertilizer to control long-term change in tundra productivity. *Ecology* **82**, 3163–3181 (2001).
15. van Wijk, M. T., Williams, M. & Shaver, G. R. Tight coupling between leaf area index and foliage N content in arctic plant communities. *Oecologia* **142**, 421–427 (2005).
16. Hobbie, S. E. & Gough, L. Foliar and soil nutrients in tundra on glacial landscapes of contrasting ages in northern Alaska. *Oecologia* **131**, 453–462 (2002).
17. Wullschleger, S. D. Biochemical Limitations to Carbon Assimilation in C3 Plants—A Retrospective Analysis of the A/Ci Curves from 109 Species. *J. Exp. Bot.* **44**, 907–920 (1993).
18. Davidson, C. D. The modeled effects of fire on carbon balance and vegetation abundance in alaska tundra. (University of Illinois at Urbana-Champaign, 2012).
19. Shaver, G. R. & Chapin, F. S. Production: Biomass Relationships and Element Cycling in Contrasting Arctic Vegetation Types. *Ecol. Monogr.* **61**, 1–31 (1991).

20. Berner, L. T. *et al.* Biomass allometry for alder, dwarf birch, and willow in boreal forest and tundra ecosystems of far northeastern Siberia and north-central Alaska. *For. Ecol. Manage.* **337**, 110–118 (2015).
21. Zona, D. & Oechel, W. FLUXNET2015 US-Atq Atqasuk, Dataset (2003-2008). doi:10.18140/FLX/1440067
22. Oechel, W. C. AmeriFlux US-HVa Happy Valley, Dataset. (1994-1995) https://doi.org/10.17190/AMF/1246064.
23. Zona, D. & Oechel, W. FLUXNET2015 US-Ivo Ivotuk, Dataset (2004-2007). https://doi.org/10.18140/FLX/1440073.
24. Schuur, E. A. & Schuur, T. AmeriFlux US-EML Eight Mile Lake Permafrost thaw gradient, Healy Alaska., Dataset. https://doi.org/10.17190/AMF/1418678. (2008).
25. Myneni, R., Knyazikhin, Y. & Park, T. MCD15A3H MODIS/Terra+Aqua Leaf Area Index/FPAR 4-day L4 Global 500m SIN Grid V006. distributed by NASA EOSDIS Land Processes DAAC, [Accessed 2019-02-17]. (2015). Available at: https://doi.org/10.5067/MODIS/MCD15A3H.006.
26. Hall, D. K., Salomonson, V. V. & Riggs, G. A. MODIS/Terra Snow Cover Daily L3 Global 500m SIN Grid, Version 6. [MOD10A1]. Boulder, Colorado USA. NASA National Snow and Ice Data Center Distributed Active Archive Center. (2016). doi:https://doi.org/10.5067/MODIS/MOD10A1.006
27. Friedl, M., J. Gray & D. Sulla-Menashe. MCD12Q2 MODIS/Terra+Aqua Land Cover Dynamics Yearly L3 Global 500m SIN Grid V006. distributed by NASA EOSDIS Land Processes DAAC [Accessed 2019-07-17]. (2019). doi:doi.org/10.5067/MODIS/MCD12Q2.006
28. Oechel, W. & Kalhori, A. ABoVE: CO2 and CH4 Fluxes and Meteorology at Flux Tower Sites, Alaska, 2015-2017. https://doi.org/10.3334/ORNLDAAC/1562. (2018).
29. Zona, D. *et al.* Light-stress avoidance mechanisms in a Sphagnum -dominated wet coastal Arctic tundra ecosystem in Alaska. *Ecology* **92**, 633–644 (2011).
30. Euskirchen, E. S. Surface carbon, water and energy fluxes measured by eddy covariance at 3 sites within the Alaska Peatlands Experiment and Bonanza Creek Experimental Forest 2010-2013, Bonanza Creek LTER - University of Alaska Fairbanks. BNZ:524, http://www.lter.uaf.edu/da. (2019). doi:10.6073/pasta/d9498c93f03043ae5940479bcbe1debd
31. Edgar, C. & Euskirchen, E. S. Surface carbon, water and energy fluxes measured by eddy covariance at 3 sites within the Alaska Peatlands Experiment and Bonanza Creek Experimental Forest 2014-2018, Bonanza Creek LTER - University of Alaska Fairbanks. BNZ:708, http://www.lter.uaf.edu/da. (2019). doi:10.6073/pasta/4fabab3846113a1866b06f1b3d6d52a3
